# The LRP1/CD91 ligands, tissue-type plasminogen activator, α_2_-macroglobulin, and soluble cellular prion protein have distinct co-receptor requirements for activation of cell-signaling

**DOI:** 10.1038/s41598-022-22498-1

**Published:** 2022-10-20

**Authors:** Elisabetta Mantuano, Pardis Azmoon, Michael A. Banki, Cory B. Gunner, Steven L. Gonias

**Affiliations:** Department of Pathology, University of California San Diego, La Jolla, CA 92093 USA

**Keywords:** Innate immunity, Cell signalling, Cytokines, Signal transduction

## Abstract

LDL Receptor-related Protein-1 (LRP1/CD91) binds diverse ligands, many of which activate cell-signaling. Herein, we compared three LRP1 ligands that inhibit inflammatory responses triggered by lipopolysaccharide (LPS), including: enzymatically-inactive tissue-type plasminogen activator (EI-tPA); activated α_2_-macroglobulin (α_2_M); and S-PrP, a soluble derivative of nonpathogenic cellular prion protein (PrP^C^). In bone marrow-derived macrophages, the N-methyl-D-aspartate receptor was essential for all three LRP1 ligands to activate cell-signaling and inhibit LPS-induced cytokine expression. Intact lipid rafts also were essential. Only α_2_M absolutely required LRP1. LRP1 decreased the EI-tPA concentration required to activate cell-signaling and antagonize LPS but was not essential, mimicking its role as a S-PrP co-receptor. Membrane-anchored PrP^C^ also functioned as a co-receptor for EI-tPA and α_2_M, decreasing the ligand concentration required for cell-signaling and LPS antagonism; however, when the concentration of EI-tPA or α_2_M was sufficiently increased, cell-signaling and LPS antagonism occurred independently of PrP^C^. S-PrP is the only LRP1 ligand in this group that activated cell-signaling independently of membrane-anchored PrP^C^. EI-tPA, α_2_M, and S-PrP inhibited LPS-induced LRP1 shedding from macrophages, a process that converts LRP1 into a pro-inflammatory product. Differences in the co-receptors required for anti-inflammatory activity may explain why LRP1 ligands vary in ability to target macrophages in different differentiation states.

## Introduction

Low-density lipoprotein (LDL) receptor-related protein-1 (LRP1/CD91) is a member of the LDL receptor family, which participates in numerous biological processes, including endocytosis, phagocytosis, antigen presentation, efferocytosis, and cell-signaling^1,2^. LRP1 binds more than 100 ligands, including apolipoprotein E-containing lipoproteins, proteases, protease inhibitor complexes, extracellular matrix proteins, growth factors, heat shock proteins, and proteins released by injured cells^3–5^. Cell-signaling responses initiated by ligands that bind to LRP1 typically require one or more co-receptors^2^; however, the composition of the receptor assemblies that initiate cell-signaling in response to LRP1 ligands remains incompletely understood.

The N-methyl-D-aspartic acid (NMDA) receptor (NMDA-R) functions in concert with LRP1 to trigger cell-signaling in response to a number of LRP1 ligands, including: tissue-type plasminogen activator (tPA), α_2_-macroglobulin (α_2_M), and a soluble derivative of non-pathogenic cellular prion protein (PrP^C^), which is similar in structure to a derivative of PrP^C^ released from cells by ADAM10 (S-PrP)^6–12^. In neuron-like cells, the NMDA-R may mediate cell-signaling in response to tPA independently of LRP1 if the tPA concentration is sufficiently increased^6^. Membrane-anchored PrP^C^ also has been described as an LRP1 co-receptor for tPA in neuron-like cells^13^; however; membrane-anchored PrP^C^ does not participate in the cellular response to S-PrP^8,14^. These results suggest that the receptor assemblies required for activation of cell-signaling by different LRP1 ligands may be distinct, depending on the ligand and the target cell. Differences in the co-receptors required may represent an important mechanism by which LRP1 ligands activate cell-signaling in a ligand-specific manner.

LRP1-activated cell-signaling regulates cell physiology. In neurons and neuron-like cells, tPA, α_2_M, and S-PrP promote neurite outgrowth and cell survival^6,8,9,15–20^. In Schwann cells, the same LRP1 ligands promote cell survival and cell migration^8,21–24^, and in macrophages, tPA, α_2_M, and S-PrP attenuate pro-inflammatory responses initiated by Pattern Recognition Receptors (PRRs)^14,25–27^. By opposing PRRs, LRP1 may regulate inflammation^26,28^.

Herein, we compared the receptor assemblies required by the LRP1 ligands, tPA, activated α_2_M, and S-PrP, for activation of cell-signaling and inhibition of lipopolysaccharide (LPS)-induced inflammatory cytokine expression in bone marrow-derived macrophages (BMDMs). For all three LRP1 ligands, the NMDA-R and intact lipid rafts were essential; the requirement for the NMDA-R was not overcome by increasing the ligand concentration. By contrast, only activated α_2_M demonstrated an absolute requirement for LRP1. LRP1 substantially decreased the concentration of tPA required to elicit biological responses in BMDMs but was not absolutely essential, as had been previously reported for S-PrP^14^. Membrane-anchored PrP^C^ functioned similarly to LRP1, decreasing the concentration of tPA and α_2_M required to elicit responses in BMDMs. The function of PrP^C^ as a co-receptor for both tPA and α_2_M distinguishes these LRP1 ligands from S-PrP, which signals independently of PrP^C^^14^. Differences in the required co-receptors may explain why macrophages in different states of differentiation respond selectively to LRP1 ligands.

## Results

### Lipid rafts are required for activation of cell-signaling by three distinct LRP1 ligands in BMDMs

**Figure 1:**
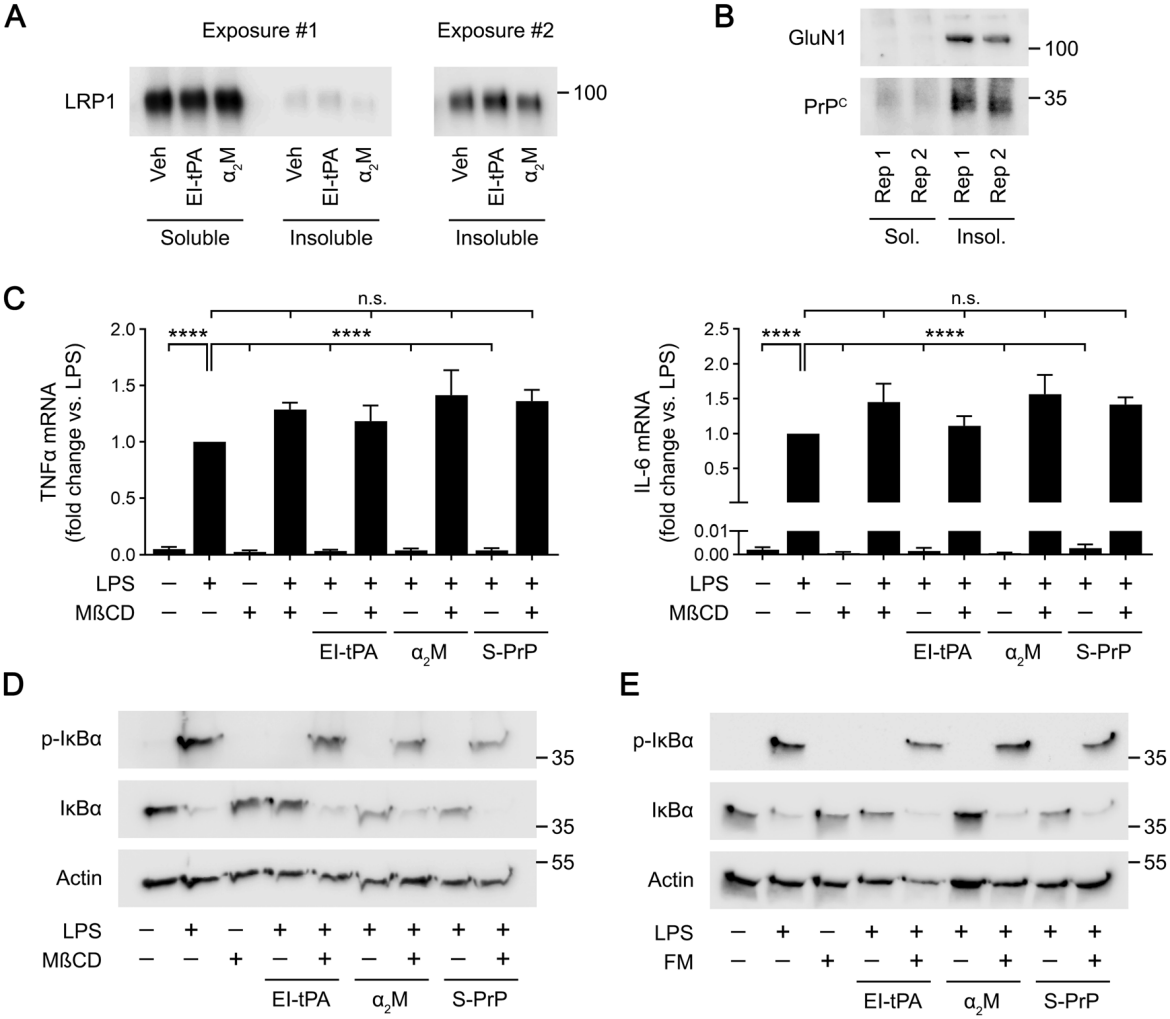
Lipid rafts are required for the anti-inflammatory activity of EI-tPA, α_2_M and S-PrP in BMDMs. (**A**) BMDMs isolated from WT mice were treated with EI-tPA (10 nM), α_2_M (10 nM) or vehicle for 1 h. Triton X-100 extracts were prepared in which cell-surface proteins were biotinylated. The biotinylated detergent-soluble and -insoluble fractions were compared by immunoblot analysis to detect LRP1. A higher exposure of the detergent-insoluble fraction is shown (Exposure #2). **(B)** Triton X-100 extracts from BMDMs isolated from WT mice were prepared in which cell-surface proteins were biotinylated. The biotinylated detergent-soluble and -insoluble fractions were compared by immunoblot analysis to detect GluN1 and PrP^C^. Two replicates (Rep 1 and 2) of each condition are shown. **(C)** BMDMs were pretreated with MβCD (1.0 mM), or vehicle for 30 min and then treated with LPS (0.1 µg/ml) and EI-tPA (10 nM), α_2_M (10 nM) or S-PrP (40 nM) for 6 h. RT-qPCR was performed to compare mRNA levels for TNFα and IL-6 (n = 4–6). Data are expressed as the mean ± SEM (One-way ANOVA; ****P < 0.0001, n.s.: not statistically significant). **(D)** BMDMs were pretreated with MβCD (1.0 mM), or vehicle for 30 min and then treated with LPS (0.1 µg/ml) and EI-tPA (10 nM), α_2_M (10 nM) or S-PrP (40 nM) for 1 h, as indicated. Cell extracts were subjected to immunoblot analysis to detect phospho-IκBα, total IκBα, and β-actin. **(E)** BMDMs were pretreated with FM (25 µM) or vehicle for 24 h and then treated with LPS (0.1 µg/ml) and EI-tPA (10 nM), α_2_M (10 nM), S-PrP (40 nM) or vehicle for 1 h, as indicated. Immunoblot analysis was performed to detect phospho-IκBα, total IκBα, and β-actin.

Only a small fraction of the LRP1 present in most studied cell types partitions into lipid rafts^29^. However, in neuron-like cells, activation of cell-signaling by EI-tPA and α_2_M requires lipid rafts^19^. To test whether LRP1 is present in lipid rafts in BMDMs, we applied a previously developed assay in which cell surface proteins are selectively labeled with a membrane-impermeable biotinylation reagent^29^. Biotinylated proteins are affinity-captured and separated into Triton X-100-soluble and -insoluble fractions; the latter includes lipid raft-associated proteins^29,30^. Figure 1A shows that in BMDMs, LRP1 is mainly localized in the Triton X-100-soluble fraction; however, a small fraction of the LRP1 partitioned into the detergent-insoluble fraction, suggesting that some LRP1 is lipid raft-associated. In cells treated for 1 h with a recombinant derivative of tPA that is enzymatically inactive but still interacts with LRP1 (EI-tPA, 10 nM) or with 10 nM activated α_2_M, the fraction of LRP1 that localized in the detergent-insoluble fraction was not significantly changed. In contrast with LRP1, the essential GluN1 subunit of the NMDA-R and PrP^C^ were identified selectively in BMDM detergent-insoluble fractions, implying localization principally to lipid rafts (Fig. 1B). Because PrP^C^ is glycosylphosphatidylinositol-anchored to the plasma membrane, it is already known to partition into lipid rafts^31–33^. The NMDA-R also has been previously identified in rafts^34–36^.

To test the role of lipid rafts in the response of BMDMs to LRP1 ligands, first BMDMs were pre-treated with methyl-β-cyclodextrin (MβCD), a cholesterol sequestration reagent that disrupts lipid rafts^37,38^. The cells were then treated with lipopolysaccharide (LPS; 0.1 µg/ml) for 6 h, in the presence and absence of EI-tPA (10 nM), activated α_2_M (10 nM), or S-PrP (40 nM). EI-tPA was studied, as opposed to wild-type tPA to avoid inadvertent activation of plasminogen, because plasmin regulates innate immunity independently of tPA^39^. The response to LPS was determined by measuring expression of the mRNAs encoding tumor necrosis factor-α (TNFα) and interleukin-6 (IL-6). In the absence of MβCD, LPS significantly increased expression of both inflammatory cytokines (Fig. 1C). The response to LPS was completely inhibited by EI-tPA (10 nM), α_2_M (10 nM), and S-PrP (40 nM), as anticipated^14,26^. MβCD did not affect expression of TNFα or IL-6 in LPS-treated or untreated cells. However, when BMDMs were pre-treated with MβCD, the activity of all three LRP1 ligands was neutralized and cytokine mRNA expression was restored to the level observed in cells treated with LPS alone.

We confirmed that MβCD inhibits the activity of LRP1 ligands in IκBα phosphorylation experiments. BMDMs were pre-treated with MβCD or vehicle and then with LPS for 1 h, in the presence and absence of EI-tPA, α_2_M, or S-PrP. In the absence of LRP1 ligands, LPS caused IκBα phosphorylation and a concomitant decrease in the abundance of total IκBα (Fig. 1D), as anticipated^27^. The response to LPS was blocked by all three LRP1 ligands. MβCD neutralized the activity of the LRP1 ligands, restoring the effects of LPS on IκBα phosphorylation and abundance.

To confirm that intact lipid rafts are required for the activity of LRP1 ligands in BMDMs, we pre-treated the cells with 25 µM fumonisin B1 (FM), a reagent that blocks synthesis of sphingolipids that are key components of lipid rafts^40^. The BMDMs were then treated with LPS (0.1 µg/ml), in the presence and absence of EI-tPA (10 nM), α_2_M (10 nM), or S-PrP (40 nM). FM blocked the ability of the LRP1 ligands to prevent LPS-induced IκBα phosphorylation and the accompanying decrease in IκBα abundance (Fig. 1E). These results demonstrate that intact lipid rafts are required for responses initiated by all three of the studied LRP1 ligands in BMDMs.

### The NMDA-R and Src family kinases are essential mediators of tPA, α_2_M, and S-PrP-activated cell-signaling in BMDMs

**Figure 2:**
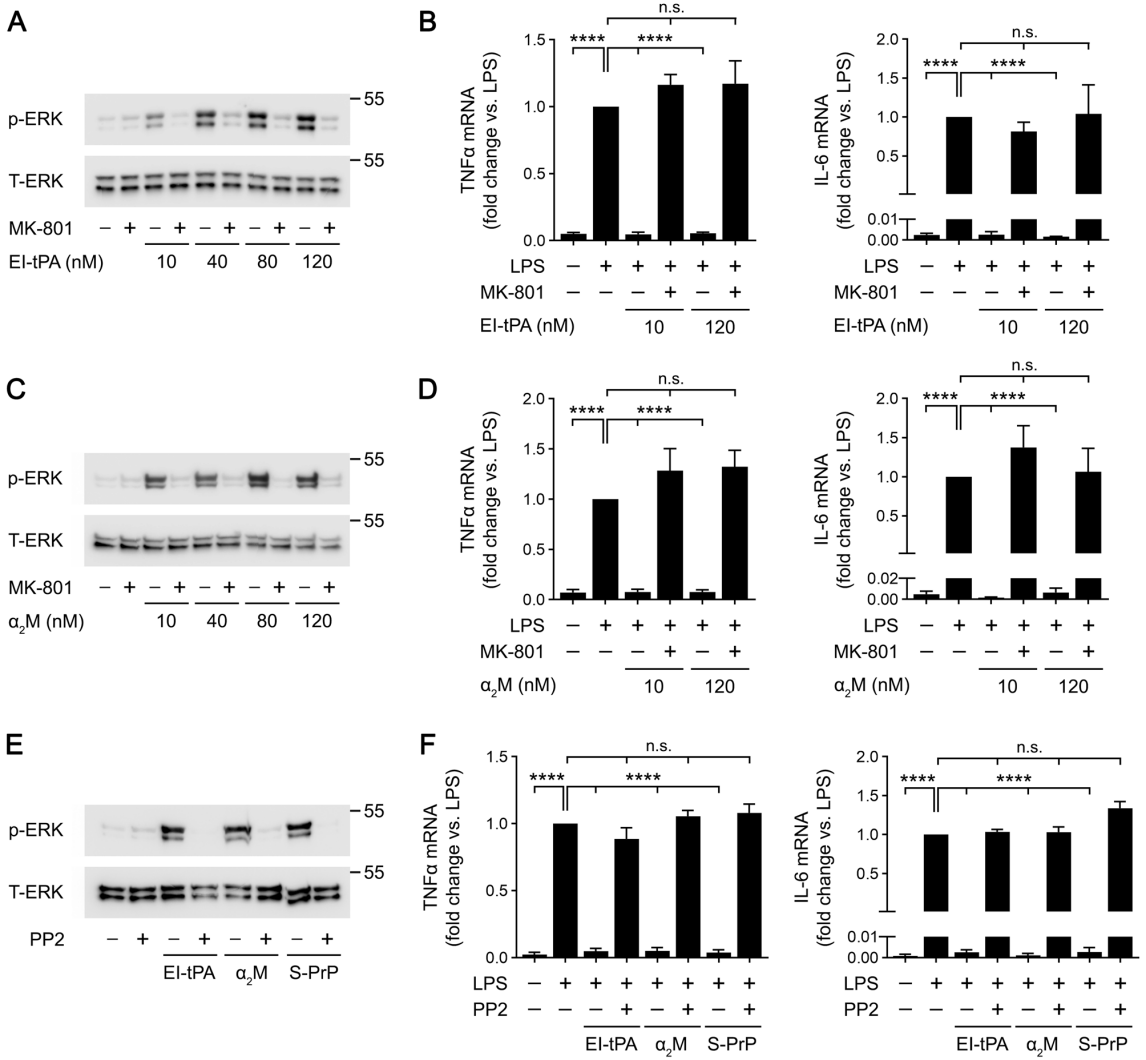
The NMDA-R and SFKs are important mediators of EI-tPA, α_2_M and S-PrP cell-signaling in macrophages. (**A**) BMDMs were pretreated with MK801 (1.0 µM), or vehicle for 30 min, and then stimulated for 1 h with increasing concentrations of EI-tPA (10–120 nM). Cell extracts were subjected to immunoblot analysis to detect phospho-ERK1/2 and total ERK1/2. **(B)** BMDMs were pretreated with MK801 (1.0 µM), or vehicle for 30 min then treated with LPS (0.1 μg/mL), LPS plus El-tPA (12 nM), LPS plus El-tPA (120 nM), or vehicle for 6 h. RT-qPCR was performed to compare mRNA levels for TNFα and IL-6 (n = 4–6). **(C)** BMDMs were pretreated with MK801 (1.0 µM), or vehicle for 30 min, and then stimulated for 1 h with increasing concentrations of α_2_M (10–120 nM). Cell extracts were subjected to immunoblot analysis to detect phospho-ERK1/2 and total ERK1/2. **(D)** BMDMs were pretreated with MK801 (1.0 µM), or vehicle for 30 min then treated with LPS (0.1 μg/mL), α_2_M (10 nM), LPS plus α_2_M, or vehicle for 6 h. RT-qPCR was performed to compare mRNA levels for TNFα and IL-6 (n = 4–5). **(E)** BMDMs were pretreated with PP2 (1.0 µM), or vehicle for 2 h, and then stimulated for 1 h with EI-tPA (10 nM), α_2_M (10 nM), S-PrP (40 nM) or vehicle. Cell extracts were subjected to immunoblot analysis to detect phospho-ERK1/2 and total ERK1/2. **(F)** BMDMs were pretreated with PP2 (1.0 µM) or vehicle for 2 h, and then with LPS (0.1 µg/ml) plus EI-tPA (10 nM), α_2_M (10 nM), S-PrP (40 nM), or vehicle for 6 h. RT-qPCR was performed to compare mRNA levels for TNFα and IL-6 (n = 4–5). Data are expressed as the mean ± SEM (One-way ANOVA; ****P < 0.0001, n.s.: not statistically significant).

BMDMs express the essential GluN1 subunit together with a number of GluN2 subunits, including predominantly GluN2A and GluN2D^24^. A tPA binding site has been identified in the N-terminal domain of the GluN1 subunit and, in cell types other than BMDMs, complexes of GluN1 with GluN2D have been implicated in tPA biological responses^12,41–44^. The NMDA-R appears to be essential for mediating S-PrP responses in BMDMs because the uncompetitive NMDA-R antagonist, Dizocilpine/MK-801, blocks the anti-LPS activity of S-PrP, even when the concentration of S-PrP is significantly increased^14^. In new studies, we treated BMDMs with MK-801 (1.0 µM) and tested the ability of these cells to respond to increasing concentrations of EI-tPA or α_2_M. In order to study BMDM responses to EI-tPA independently of LPS, we examined ERK1/2 activation. Figure 2A shows that, in the absence of MK-801, ERK1/2 activation was apparent throughout the studied EI-tPA concentration range (10–120 nM). MK-801 blocked ERK1/2 activation by EI-tPA and the effects of MK-801 were not overcome by increasing the EI-tPA concentration.

Supporting results were obtained when we studied the activity of EI-tPA as an inhibitor of LPS-induced cytokine expression in BMDMs. In the absence of MK-801, 10 nM EI-tPA and 120 nM EI-tPA were equally effective at inhibiting the effects of LPS on expression of TNFα and IL-6 (Fig. 2B). The activity of EI-tPA as an inhibitor of LPS-induced cytokine expression was blocked by MK-801 and the effects of MK-801 were not overcome by the higher EI-tPA concentration.

Next, we studied activated α_2_M. ERK1/2 phosphorylation was observed in BMDMs treated with increasing concentrations of α_2_M, from 10 to 120 nM. The response to α_2_M was neutralized by MK-801, irrespective of the α_2_M concentration (Fig. 2C). In BMDMs treated with LPS, α_2_M at 10 nM or 120 nM blocked expression of TNFα and IL-6 (Fig. 2D). Again, the activity of α_2_M was inhibited by MK-801 irrespective of the α_2_M concentration. These results suggest that the NMDA-R is essential for macrophages to respond to EI-tPA and activated α_2_M, as was previously observed with S-PrP^14^.

Src family kinases (SFKs) have been described as important upstream mediators of LRP1 signaling events^8,14,45^. SFK activation occurs downstream of the NMDA-R, apparently in caveolin-1-containing lipid rafts^46^. Figure 2E shows that the SFK inhibitor, PP2, blocked ERK1/2 phosphorylation in BMDMs treated with EI-tPA, α_2_M, and S-PrP. Similarly, PP2 blocked the ability of EI-tPA, α_2_M, and S-PrP to antagonize LPS-induced cytokine expression (Fig. 2F). Thus, SFKs serve as conserved upstream mediators of the anti-inflammatory activity of the three LRP1 ligands under investigation here.

### Membrane-anchored PrP^C^ and LRP1 facilitate but are not required for the macrophage response to EI-tPA

**Figure 3:**
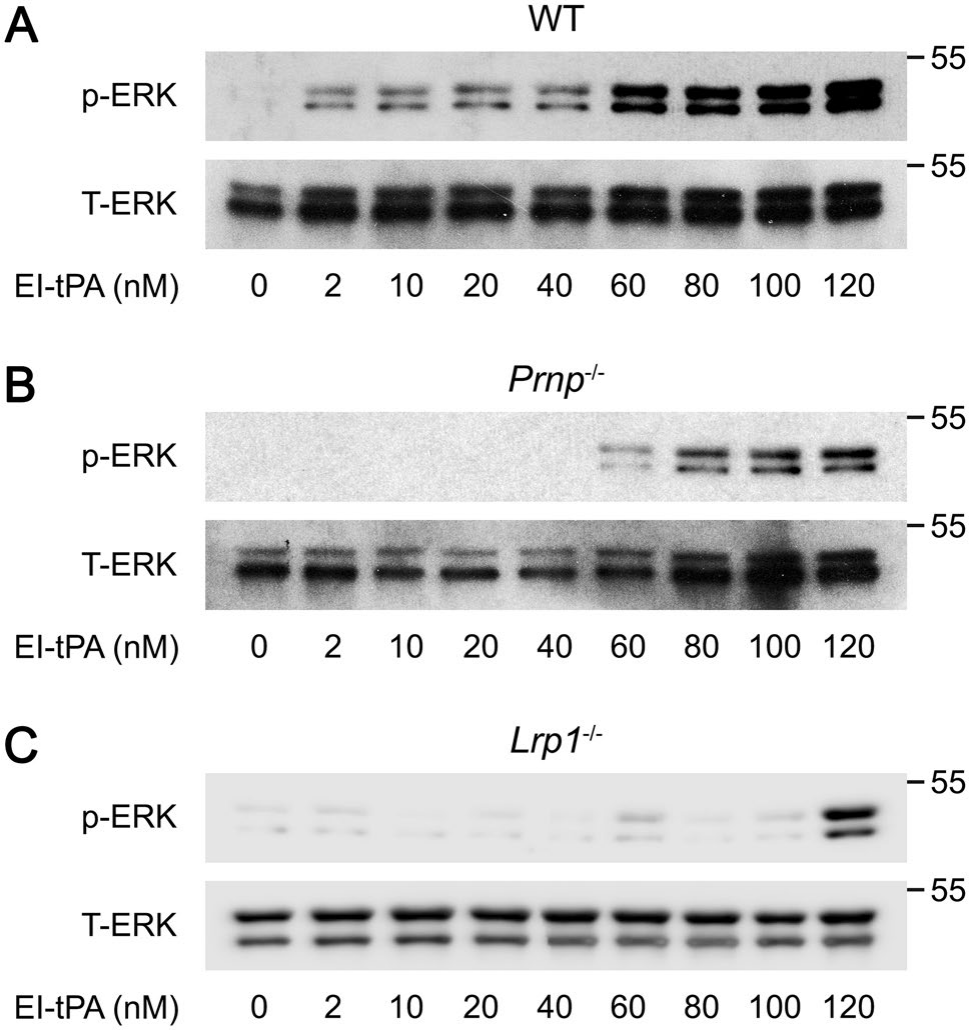
Deletion of the genes encoding membrane-anchored PrP^C^ or LRP1 in BMDMs decreases the concentration of EI-tPA required for activation of ERK1/2. (**A**) BMDMs isolated from WT mice were treated for 1 h with increasing concentrations of EI-tPA (2–120 nM). Cell extracts were subjected to immunoblot analysis to detect phospho-ERK1/2 and total ERK1/2. **(B)** PrP^C^-deficient BMDMs isolated from *Prnp*^*−/−*^ mice were treated for 1 h with increasing concentrations of EI-tPA (2–120 nM). Cell extracts were subjected to immunoblot analysis to detect phospho-ERK1/2 and total ERK1/2. **(C)** LRP1-deficient BMDMs from m*Lrp1*^−/−^ mice were treated for 1 h with increasing concentrations of EI-tPA (2–120 nM). Cell extracts were subjected to immunoblot analysis to detect phospho-ERK1/2 and total ERK1/2.

To study the function of LRP1 and PrP^C^ as EI-tPA co-receptors in macrophages, we harvested BMDMs from wild-type (WT) mice, from *Prnp*^−/−^ mice in which the gene encoding PrP^C^ is globally deleted, and from *mLRP1*^−/−^ mice, in which *Lrp1* is deleted in myeloid cells including monocytes and macrophages. Figure 3A shows that in WT BMDMs, EI-tPA at concentrations from 2.0 to 120 nM activated ERK1/2, as previously reported^26^. EI-tPA also activated ERK1/2 in PrP^C^-deficient BMDMs isolated from *Prnp*^−/−^ mice; however, the minimum concentration of EI-tPA required to activate ERK1/2 was increased to about 60 nM (Fig. 3B). In LRP1-deficient BMDMs harvested from m*Lrp1*^−/−^ mice, EI-tPA reproducibly activated ERK1/2 only when the EI-tPA concentration was increased to 120 nM (Fig. 3C). These results suggest that although membrane-anchored PrP^C^ and LRP1 facilitate EI-tPA signaling in BMDMs, these receptors are not essential and their requirement may be overcome by increasing the EI-tPA concentration.

**Figure 4:**
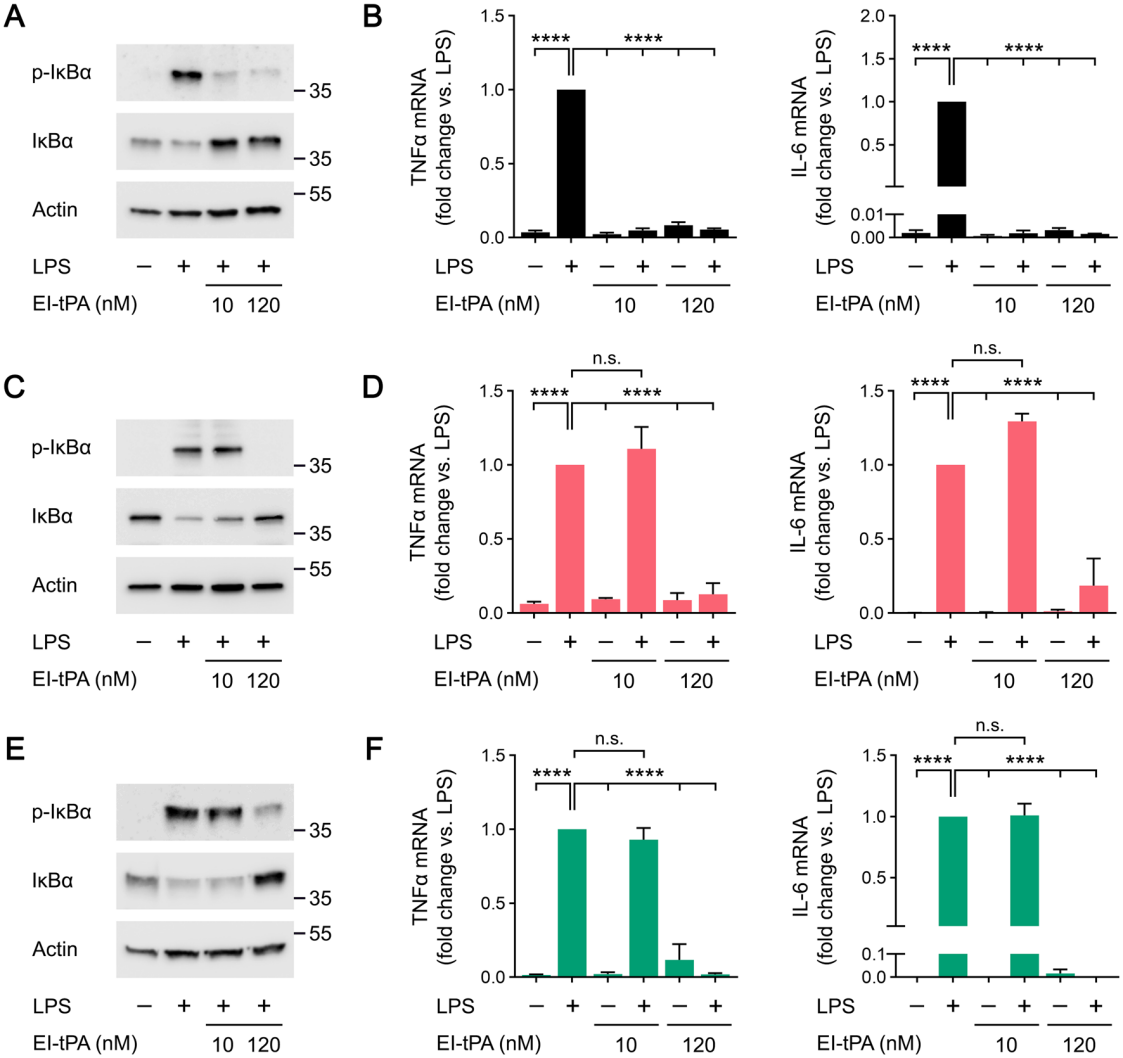
The anti-inflammatory activity of EI-tPA is facilitated by membrane-anchored PrP^C^ and LRP1. (**A**) BMDMs isolated from WT mice were treated for 1 h with LPS (0.1 μg/mL), LPS plus 10 nM El-tPA, LPS plus 120 nM El-tPA, or vehicle. Cell extracts were subjected to immunoblot analysis to detect phospho-IκBα, total IκBα, and β-actin. **(B)** WT BMDMs were treated with LPS (0.1 μg/mL), LPS plus 10 nM El-tPA, LPS plus 120 nM El-tPA, or vehicle for 6 h. RT-qPCR was performed to compare mRNA levels for TNFα and IL-6 (n = 4–6). **(C)** PrP^C^-deficient BMDMs isolated from *Prnp*^*−/−*^ mice were treated for 1 h with LPS (0.1 μg/mL), LPS plus 10 nM El-tPA, LPS plus 120 nM El-tPA, or vehicle. Cell extracts were subjected to immunoblot analysis to detect phospho-IκBα, total IκBα, and β-actin. **(D)** PrP^C^-deficient BMDMs were treated with LPS (0.1 μg/mL), LPS plus 10 nM El-tPA, LPS plus 120 nM El-tPA, or vehicle for 6 h. RT-qPCR was performed to compare mRNA levels for TNFα and IL-6 (n = 4–5). **(E)** LRP1-deficient BMDMs isolated from *mLrp1*^*−/−*^ mice were treated for 1 h with LPS (0.1 μg/mL), LPS plus 10 nM El-tPA, LPS plus 120 nM El-tPA, or vehicle. Cell extracts were subjected to immunoblot analysis to detect phospho-IκBα, total IκBα, and β-actin. **(F)** LRP1-deficient BMDMs were treated with LPS (0.1 μg/mL), LPS plus 10 nM El-tPA, LPS plus 120 nM El-tPA, or vehicle for 6 h. RT-qPCR was performed to compare mRNA levels for TNFα and IL-6 (n = 4–5). Data are expressed as the mean ± SEM (One-way ANOVA; ****P < 0.0001, n.s.: not statistically significant).

To further test our model regarding the activity of membrane-anchored PrP^C^ and LRP1 as mediators of EI-tPA responses in macrophages, we studied the ability of EI-tPA to block the response to LPS. Figure 4A shows that, in WT BMDMs, EI-tPA blocked LPS-induced IκBα phosphorylation equally well when the EI-tPA concentration was 10 nM or 120 nM. 10 nM EI-tPA and 120 nM EI-tPA also were equally effective at blocking LPS-induced expression of TNFα and IL-6 (Fig. 4B).

In PrP^C^-deficient BMDMs, 120 nM EI-tPA blocked LPS-induced IκBα phosphorylation; however, 10 nM EI-tPA was ineffective (Fig. 4C). Similarly, 120 nM EI-tPA inhibited expression of TNFα and IL-6 in LPS-treated PrP^C^-deficient BMDMs; however, 10 nM EI-tPA was ineffective (Fig. 4D).

In LRP1-deficient BMDMs, 10 nM EI-tPA failed to inhibit LPS-induced IκBα phosphorylation whereas 120 nM EI-tPA at least partially blocked the LPS response (Fig. 4E). Similarly, in cytokine mRNA expression studies in LRP1-deficient BMDMs, 10 nM EI-tPA did not have a significant effect whereas 120 nM EI-tPA significantly inhibited the LPS response (Fig. 4F). Taken together, these results demonstrate that in BMDMs, neither membrane-anchored PrP^C^ nor LRP1 is essential for cells to respond to EI-tPA; however, both plasma membrane proteins significantly decrease the concentration of EI-tPA required for efficacy.

### ***LRP1 is required for and PrP***^***C***^*** facilitates the BMDM response to activated α***_***2***_***M***

**Figure 5:**
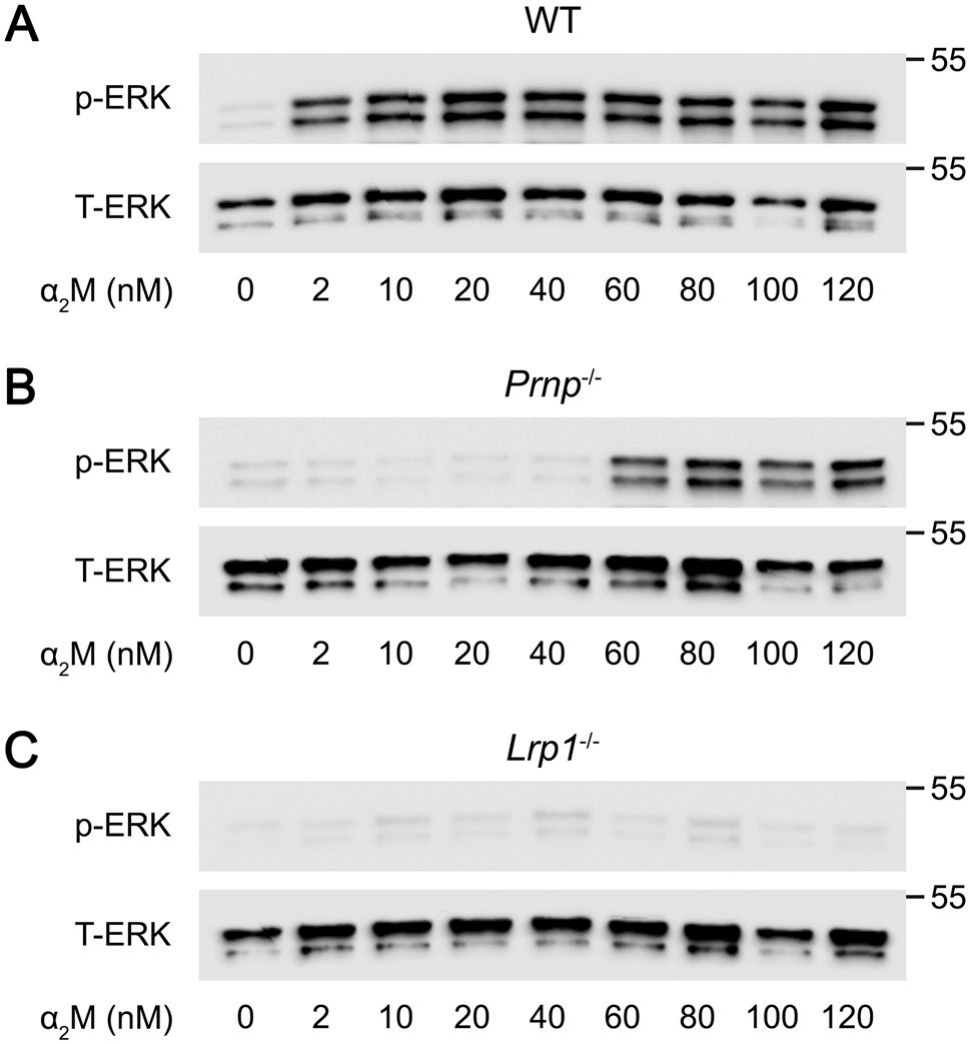
Membrane-anchored PrP^C^ facilitates and LRP1 is essential for activation of ERK1/2 by α_2_M in BMDMs. (**A**) BMDMs isolated from WT mice were treated for 1 h with increasing concentrations of activated α_2_M (2–120 nM). Cell extracts were subjected to immunoblot analysis to detect phospho-ERK1/2 and total ERK1/2. **(B)** PrP^C^-deficient BMDMs isolated from *Prnp*^*−/−*^ mice were treated for 1 h with increasing concentrations of activated α_2_M (2–120 nM). Cell extracts were subjected to immunoblot analysis to detect phospho-ERK1/2 and total ERK1/2. **(C)** LRP1-deficient BMDMs isolated from m*Lrp1*^−/−^ mice were treated for 1 h with increasing concentrations of activated α_2_M (2–120 nM). Cell extracts were subjected to immunoblot analysis to detect phospho-ERK1/2 and total ERK1/2.

Treatment of WT BMDMs with activated α_2_M, at concentrations from 2.0 to 120 nM, in the absence of LPS, activated ERK1/2 (Fig. 5A). In PrP^C^-deficient BMDMs harvested from *Prnp*^−/−^ mice, the concentration of α_2_M required to activate ERK1/2 was increased to 60 nM (Fig. 5B). In LRP1-defiicent BMDMs, ERK1/2 activation was not observed throughout the α_2_M concentration range studied (Fig. 5C).

**Figure 6:**
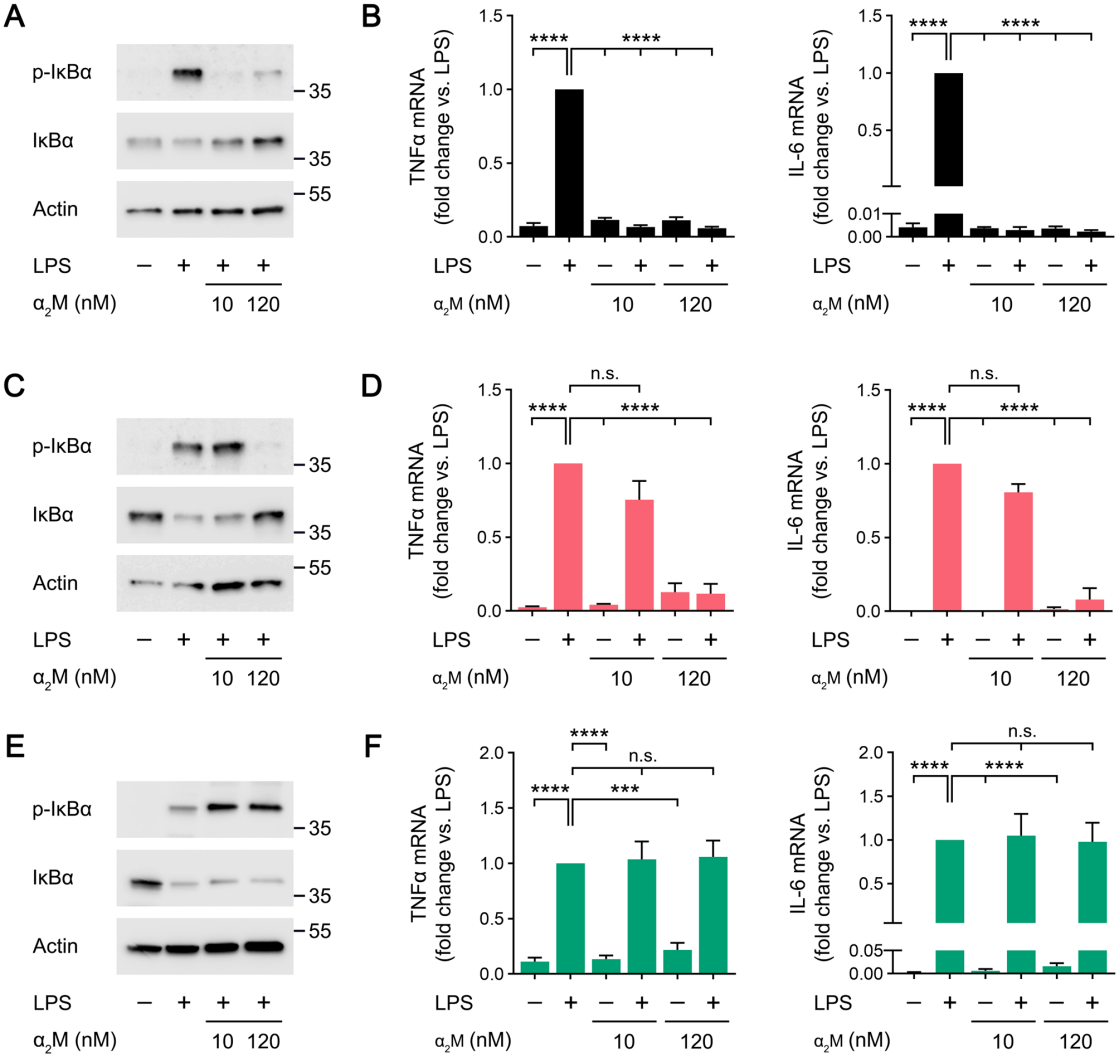
The anti-inflammatory activity of activated α_2_M is facilitated by target cell PrP^C^ and demonstrates an absolute requirement for LRP1. (**A**) BMDMs isolated from WT mice were treated for 1 h with LPS (0.1 μg/mL), LPS plus 10 nM α_2_M, LPS plus 120 nM α_2_M, or vehicle. Cell extracts were subjected to immunoblot analysis to detect phospho-IκBα, total IκBα, and β-actin. **(B)** WT BMDMs were treated with LPS (0.1 μg/mL), LPS plus 10 nM α_2_M, LPS plus 120 nM α_2_M, or vehicle for 6 h. RT-qPCR was performed to compare mRNA levels for TNFα and IL-6 *(*n = 7). **(C)** PrP^C^-deficient BMDMs isolated from *Prnp*^*−/−*^ mice were treated for 1 h with LPS (0.1 μg/mL), LPS plus 10 nM α_2_M, LPS plus 120 nM α_2_M, or vehicle. Cell extracts were subjected to immunoblot analysis to detect phospho-IκBα, total IκBα, and β-actin. **(D)** PrP^C^-deficient BMDMs were treated with LPS (0.1 μg/mL), LPS plus 10 nM α_2_M, LPS plus 120 nM α_2_M, or vehicle for 6 h. RT-qPCR was performed to compare mRNA levels for TNFα and IL-6 (n = 4). **(E)** BMDMs isolated from *mLrp1*^*−/−*^ mice were treated for 1 h with LPS (0.1 μg/mL), LPS plus 10 nM α_2_M, LPS plus 120 nM α_2_M, or vehicle. Cell extracts were subjected to immunoblot analysis to detect phospho-IκBα, total IκBα, and β-actin. **(F)** LRP1-deficient BMDMs were treated with LPS (0.1 μg/mL), LPS plus 10 nM α_2_M, LPS plus 120 nM α_2_M, or vehicle for 6 h. RT-qPCR was performed to compare mRNA levels for TNFα and IL-6 (n = 4). Data are expressed as the mean ± SEM (One-way ANOVA; ***P < .001, ****P < 0.0001, n.s.: not statistically significant).

Next, we studied the activity of α_2_M as an antagonist of LPS responses in BMDMs. In WT BMDMs, 10 nM α_2_M and 120 nM α_2_M blocked the increase in IκBα phosphorylation and the accompanying decrease in IκBα abundance induced by LPS (Fig. 6A). α_2_M, at 10 or 120 nM, also completely blocked LPS-induced expression of TNFα and IL-6 (Fig. 6B).

In PrP^C^-deficient macrophages, 10 nM α_2_M failed to block LPS-induced IκBα phosphorylation (Fig. 6C) and the increase in expression of TNFα and IL-6 caused by LPS (Fig. 6D). The activity of α_2_M was restored when its concentration was increased to 120 nM. In studies with LRP1-deficient BMDMs, α_2_M failed to inhibit LPS-induced IκBα phosphorylation even when the α_2_M concentration was 120 nM (Fig. 6E). Similarly, α_2_M at 10 and 120 nM failed to inhibit expression of TNFα and IL-6 in LPS-treated LRP1-deficient BMDMs (Fig. 6F). Thus, within the limits of the α_2_M concentration range studied, α_2_M demonstrates an absolute requirement for LRP1 as a cell-signaling receptor in BMDMs.

### Anti-inflammatory LRP1 ligands prevent LRP1 shedding

In response to LPS, the level of functional, cell-surface LRP1 in macrophages is decreased^47^. Although the decrease may partially reflect altered gene transcription^48^, shedding of LRP1 from the cell surface is a major event activated by LPS^49^. This is important because, in addition to decreasing the capacity of the cell to respond to anti-inflammatory ligands such as α_2_M, shedding of LRP1 generates a soluble derivative that is highly pro-inflammatory^49–51^.

**Figure 7:**
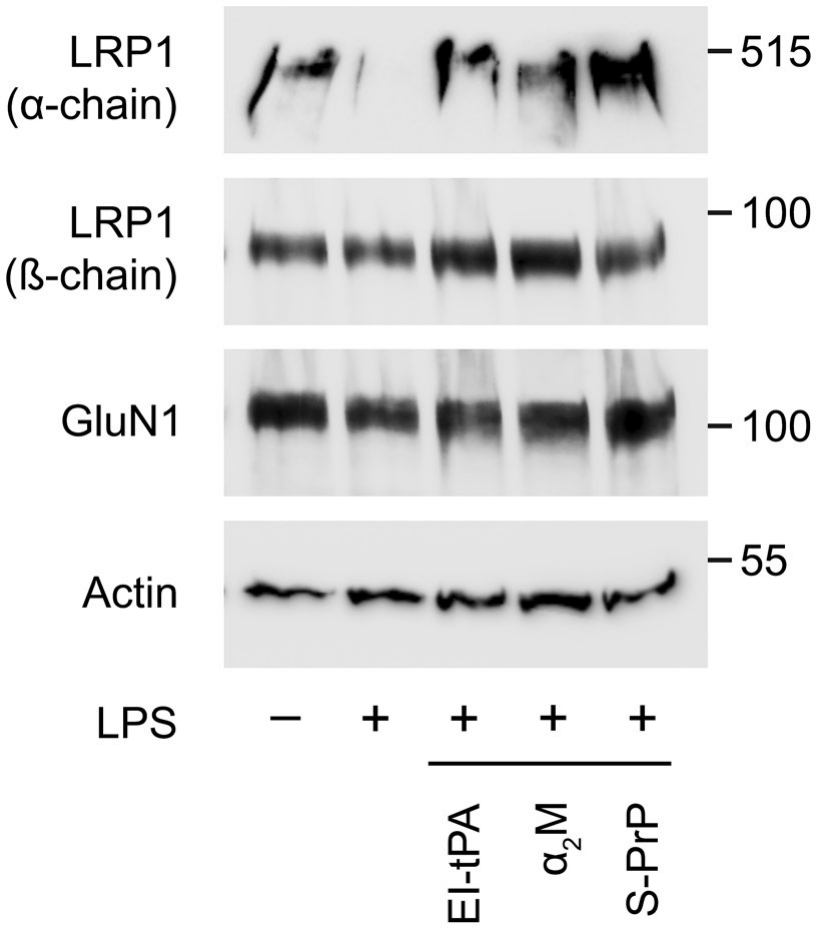
EI-tPA, α_2_M, and S-PrP prevent the decrease in cellular LRP1 α-chain caused by LPS. BMDMs were treated with LPS (0.1 µg/ml) alone and together with EI-tPA (10 nM), α_2_M (10 nM), or S-PrP (40 nM) for 6 h. Control cells were treated with vehicle. Cell extracts were subjected to immunoblot analysis to detect LRP1 α-chain, LRP1 β-chain, NMDA-R GluN1, and β-actin.

LRP1 is shed by proteolytic cleavage of the ectodomain region of the transmembrane LRP1 β-chain, causing concomitant loss of the entire 515-kDa α-chain^52^. To examine LRP1 loss from BMDMs, we used a paired immunoblotting approach, including antibodies specific for the α-chain and a separate antibody that recognizes an intracellular epitope of the β-chain, retained when LRP1 is cleaved to induce shedding^50^. Figure 7 shows that treatment of BMDMs with LPS (0.1 µg/mL) for 6 h substantially decreased LRP1 α-chain in association with the cells, without decreasing the abundance of the intracellular β-chain epitope, a result attributable to LRP1 shedding^49–52^. Figure 7 also shows, for the first time, that EI-tPA, activated α_2_M, and S-PrP prevented the LPS-induced loss of LRP1 from BMDMs. By contrast, the total abundance of NMDA-R GluN1 in BMDMs was not altered by LPS or by the LRP1 ligands. We conclude that in addition to antagonizing PRR responses, the three LRP1 ligands attenuate a pro-inflammatory pathway involving LRP1 shedding.

## Discussion

LRP1 binds over 100 ligands, including proteins released by injured and dying cells, which enables this receptor to recognize tissue injury and mediate appropriate cellular responses^3,4^. Not all LRP1 ligands activate cell-signaling and there is evidence that the signaling pathways activated may be ligand-dependent^6^. The three ligands studied here: EI-tPA; activated α_2_M; and S-PrP have seemingly overlapping effects on cells despite the lack of structural similarity. All three ligands promote neurite outgrowth in neurons, cell survival in Schwann cells, and inhibit responses triggered by TLRs in BMDMs. Activated α_2_M was amongst the first LRP1 ligands identified^53^. The role of LRP1 as a tPA receptor was first demonstrated in studies that implicated hepatic LRP1 in tPA clearance from the plasma^54^. We characterized the soluble PrP^C^ derivative, S-PrP, as an LRP1 ligand capable of activating cell-signaling^8,14^, however, others had previously demonstrated functional interactions involving LRP1 and membrane-anchored PrP^C^. LRP1 plays an essential role in PrP^C^ endocytosis^55^ and also may be involved in PrP^C^ trafficking through the biosynthetic pathway^56^. Furthermore, in neuroblastoma cells, membrane-anchored PrP^C^ was identified as an essential LRP1 co-receptor, required for tPA signaling^13^.

We now show that in BMDMs, all three LRP1 ligands, at the concentrations studied, demonstrate an absolute requirement for the NMDA-R, for lipid rafts, and SFKs in order to activate ERK1/2 and inhibit LPS responses. Our results regarding the NMDA-R are consistent with a body of recent results demonstrating the ability of the NMDA-R to serve as an activator of cell-signaling in response to protein ligands in addition to its better characterized role as an ionotropic glutamate receptor^6–9,14,24,26,57^. α_2_M was the only protein that also demonstrated an absolute requirement for LRP1. In studies with EI-tPA and S-PrP, LRP1 facilitated responses observed in BMDMs, decreasing the ligand concentration required to activate ERK1/2 or antagonize LPS; however, the requirement for LRP1 was eliminated when the concentration of EI-tPA or S-PrP was sufficiently high. This result is consistent with a model in which LRP1 functions to sequester protein ligands and deliver these ligands to the NMDA-R to activate cell-signaling. Both tPA and PrP^C^ have been reported to directly associate with the NMDA-R, independently of LRP1^44,58,59^, explaining why cellular responses may still be observed in the absence of LRP1 if the ligand concentration is sufficiently high.

**Figure 8:**
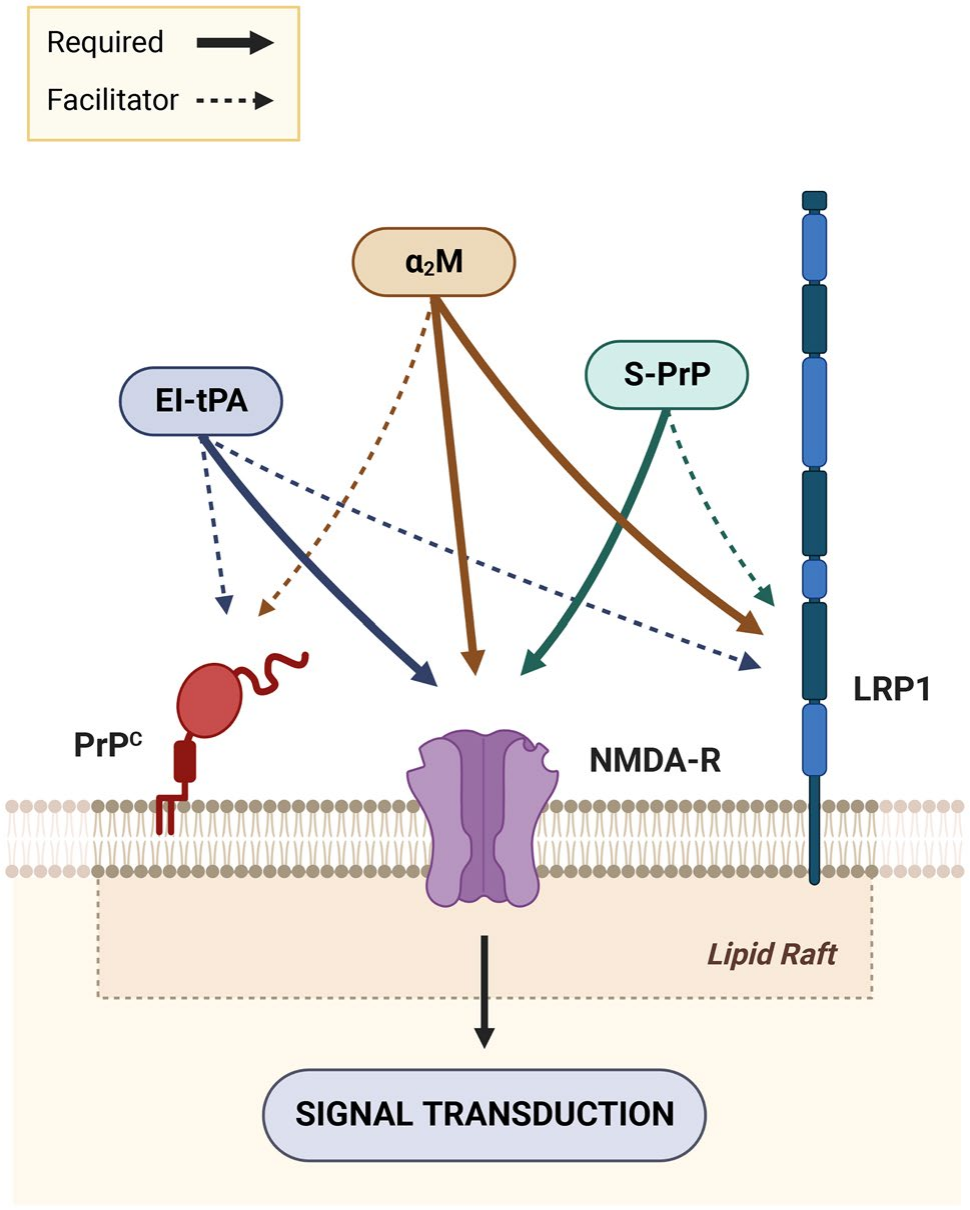
The LRP1 ligands: EI-tPA, α_2_M, and S-PrP require distinct receptor assemblies for activation of cell-signaling and regulation of LPS responses in BMDMs. The figure was created using BioRender.com.

We confirmed a functional role for membrane-anchored PrP^C^ as a co-receptor for tPA signaling, as originally proposed by Mattei et al.^13^; however, we also show that PrP^C^ is not absolutely essential for tPA-signaling and may be overcome by increasing the tPA concentration. Previous studies demonstrating high affinity binding of tPA to PrP^C^ (K_D_ of 2.5 nM)^60^ support a model in which PrP^C^ functions similarly to LRP1, sequestering tPA and presenting it to the NMDA-R for activation of cell-signaling. Our results also implicate membrane-anchored PrP^C^ as a facilitator of responses mediated by α_2_M in BMDMs. S-PrP is the only LRP1 ligand, amongst the three studied, that apparently signals with equal efficacy in BMDMs in the presence or absence of membrane-anchored PrP^C^^12^. Figure 8 summarizes how the NMDA-R, LRP1, and membrane anchored PrP^C^ differentially assemble to form receptor complexes that respond to EI-tPA, α_2_M, and S-PrP in BMDMs.

Differences in how LRP1 and PrP^C^ function as co-receptors with the NMDA-R to mediate responses to α_2_M, EI-tPA, and S-PrP may explain why different cell types or a single cell type in distinct states of differentiation respond differentially to these ligands. For example, whereas BMDMs, which are highly differentiated due to treatment with m-CSF for 7 days, respond to both EI-tPA and S-PrP, un-elicited mouse peritoneal macrophages, which are not m-CSF-treated, respond selectively to S-PrP and not EI-tPA^14,25^. The results presented here raise the hypothesis that peritoneal macrophages may have a lower level of cell-surface PrP^C^, which is required for efficient tPA-activated cell-signaling but not for S-PrP-activated cell-signaling.

There is evidence that other plasma membrane proteins also may participate in the receptor assemblies that mediate responses to LRP1 ligands. For example, in PC12 cells, cerebellar granular neurons, and in dorsal root ganglia in vivo, Trk receptors are transactivated by SFKs downstream of the LRP1-NMDA-R receptor assembly in response to the receptor binding domain of α_2_M and tPA. Similar results have been obtained with S-PrP^8,45^. Receptor tyrosine kinase (RTK) transactivation events do not require that the RTK physical associates with the receptor system that is initially activated^61^. Similarly, the EGF Receptor (EGF-R) has been implicated as an important mediator of the biological activities of tPA in neurons^62^. The role of the EGF-R may reflect direct association of tPA with the EGF-R, association of the EGF-R with the NMDA-R, or EGF-R transactivation by SFKs, similarly to Trk^63^. Myelin-associated proteins are LRP1 ligands, which recruit p75^NTR^ into a receptor assembly with LRP1 to activate cell-signaling^64^. The signaling pathways activated downstream of the LRP1-p75^NTR^ complex are distinct from those activated independently of p75^NTR^^6,64^. Glucose-regulated protein-78 (grp78), which is best known for its function as an endoplasmic reticulum (ER) chaperone^65^, may be secreted and associate with cell surfaces, where it serves as a second receptor for activated α_2_M, capable of inducing diverse effects on macrophages and cancer cells^66,67^. Although LRP1 and cell-surface grp78 may function separately as mediators of α_2_M responses, it is also possible that these proteins function in synergy to determine cellular affinity for α_2_M, as reported here for PrP^C^. Grp78 also has been identified as a cell-surface receptor for tPA, capable of regulating cell signaling^68^.

In addition to the assortment of available co-receptors, the activity of the LRP1-NMDA-R receptor assembly may be controlled by variations in the structure of the ligands, not studied in depth here. For example, whereas, in this study, we examined activated α_2_M, the majority of the α_2_M in plasma is in the native conformation, which does not interact with LRP1^69^. EI-tPA is an enzymatically-inactive single-chain variant of tPA. In previous studies, we demonstrated that a mainly two-chain variant of tPA, which is enzymatically-active, and EI-tPA regulate ERK1/2 activation and LPS responses in BMDMs equivalently^26,39,70^; however, there is evidence that in some model systems, single-chain tPA and two-chain tPA may engage cellular receptors differentially and thereby differentially regulate cell physiology^62,71,72^. Understanding variations in LRP1 ligand structure that impact the collection of receptors involved in mediating changes in cell physiology in response to ligand-binding remains an important problem.

LRP1 itself may be anti-inflammatory or pro-inflammatory. The key reaction that converts LRP1 into a pro-inflammatory agent appears to be shedding, which is mediated by proteases in the ADAM family^49–51^. The ability of agents like LPS to decrease cell-surface LRP1^47^ is reasonably explained by the fact that cell-surface LRP1 attenuates LPS responses. We have now shown that EI-tPA, α_2_M, and S-PrP preserve cell-surface LRP1 in the presence of LPS, apparently by preventing LRP1 shedding. This conserved activity of EI-tPA, α_2_M, S-PrP may represent a novel mechanism by which these proteins inhibit inflammation.

Inflammation creates complex microenvironments for LRP1-expressing macrophages and these cells participate variably in supporting the pro-inflammatory state or in tissue repair, depending on their differentiation state^14,25^. Undoubtedly, macrophages are exposed to numerous ligands for PRRs and LRP1, capable of regulating macrophage physiology. Our results support a model in which diverse plasma membrane proteins may determine the capacity of the macrophage to respond to specific LRP1 ligands.

## Materials and methods

### Ethics statement

All animal experiments performed were approved by the Institutional Animal Care and Use Committee (IACUC) of University of California San Diego (UCSD) and was conducted strictly under the guidelines for animal experimentation of UCSD (IACUC Protocol: S04074), and the ARRIVE guidelines. We have complied with all relevant ethical regulations for animal testing and research.

Human plasma was obtained from the UCSD transfusion service. The use of human plasma for protein purification was approved by Institutional Review Board at UCSD (IRB Project: 091330). All methods were carried out in accordance with relevant guidelines and regulations. In brief, de-identified fresh frozen plasma was provided by the UCSD blood bank, after the plasma was determined to be no longer necessary for patient care and would otherwise be discarded. These plasma samples were originally obtained from volunteer donors following the complete consenting process required by the Association for the Advancement of Blood and Biotherapies (AABB) guidelines.

### Proteins and reagents

S-PrP (residues 23–231 from the structure of full-length mouse PrP^C^) was expressed and purified as previously described^8^. In brief, S-PrP was expressed in *E. coli* BL21 as a His-tagged protein, which was recovered from inclusion bodies, purified by Ni^2+^-affinity chromatography, oxidized, and refolded out of denaturant. The N-terminal poly-His tail was dissociated with thrombin, which was subsequently removed by ion exchange chromatography. S-PrP preparations were > 98% pure, as determined by SDS-PAGE with silver staining and by LC–MS/MS analysis of tryptic peptides^8^. All S-PrP preparations were processed through high-capacity endotoxin removal columns (Pierce) and determined to be endotoxin-free using an endotoxin detection kit (Thermo Fisher Scientific).

Human EI-tPA, which carries the S478A mutation and thus lacks catalytic activity, and a second mutation (R275E) so the protein remains in single-chain form, was from Molecular Innovations. α_2_M was purified from human plasma and activated for binding to LRP1 by reaction with methylamine as previously described^73^. All α_2_M preparations were determined to be endotoxin-free. LPS serotype 055:B5 from *E. coli* was from Sigma-Aldrich. MK-801 and FM were from Cayman Chemicals. MβCD was from Sigma-Aldrich. PP2 was from Abcam.

### Animals

WT C57BL/6 J mice were obtained from Jackson Laboratory. To generate mice in which BMDMs are LRP1 deficient (m*Lrp1*^−/−^ mice), *Lrp1*^flox/flox^ mice were bred with mice that express Cre recombinase under the control of the lysozyme-M promoter (LysM-Cre), in the C57BL/6 J background, as previously described^74^. For experiments with macrophages harvested from m*Lrp1*^−/−^ mice, control cells were harvested from littermates that were LRP1^flox/flox^ but LysM-Cre-negative (m*Lrp1*^+/+^ mice). The *Prnp*^ZH3/ZH3^ strain of *Prnp*^−/−^ mice in the C57BL/6 background^75^ was generously provided by Dr. Adriano Aguzzi (University Hospital of Zurich, Zurich, Switzerland). The absence of mRNA encoding LRP1 and PrP^C^ in BMDMs harvested from m*Lrp1*^−/−^ and *Prnp*^−/−^ mice, respectively, was determined by RT-qPCR.

### Cell culture

BMDMs were harvested from 16-week-old male mice, as previously described^27^. Briefly, bone marrow cells were flushed from mouse femurs and plated in non-tissue culture-treated dishes. Cells were cultured in DMEM/F-12 medium containing 10% fetal bovine serum (FBS) and 20% L929 cell-conditioned medium or 20 nM recombinant mouse macrophage colony-stimulating factor (M-CSF; BioLegend) for 7 days. Non-adherent cells were eliminated. Adherent cells included > 95% BMDMs, as determined by F4/80 and CD11b immunoreactivity. Experiments were performed in medium that contained 50 µM glutamate.

### Cell signaling

Cells were transferred to serum-free medium (SFM) for 30 min and then treated with various proteins and reagents, alone or simultaneously as noted, including: LPS (0.1 µg/ml); S-PrP (40 nM); EI-tPA (10–120 nM); activated α_2_M (10–120 nM); MK-801 (1.0 µM); MβCD (1.0 mM); FM (25 µM); or vehicle (20 mM sodium phosphate, 150 mM NaCl, pH 7.4, PBS).

Cells were rinsed with ice-cold PBS and proteins were extracted in RIPA buffer (20 mM sodium phosphate, 150 mM NaCl, pH 7.4, 1% Triton X-100, 0.5% sodium deoxycholate, 0.1% SDS) supplemented with protease and phosphatase inhibitors (Thermo Fisher Scientific). Equal amounts of protein, as determined using the detergent-compatible (DC) Protein Assay (Bio-Rad), were subjected to SDS-PAGE and electro-transferred to polyvinylidene fluoride membranes. The membranes were blocked with 5% nonfat dried milk and then incubated with primary antibodies from Cell Signaling Technology that recognize: phospho-ERK1/2, total ERK1/2, phospho-IκBα, total IκBα, and β-actin. The membranes were washed and incubated with horseradish peroxidase-conjugated secondary antibody (Jackson ImmunoResearch). Immunoblots were developed using Radiance, Radiance Q, and Radiance Plus chemiluminescent substrates (Azure Biosystems) and imaged using the Azure Biosystems c300 digital system or Blue Devil autoradiography film (Genesee Scientific). Images were processed with Adobe Photoshop 23.3.2. The presented results are representative of at least three independent experiments. Uncropped blots are presented in Supplementary Figures online.

### RT-qPCR

Cells were transferred to SFM for 30 min and then treated with various proteins and reagents for 6 h. RNA was isolated using the NucleoSpin RNA kit (Macherey–Nagel) and reverse-transcribed using the iScript cDNA synthesis kit (Bio-Rad). qPCR was performed using TaqMan gene expression products (Thermo Fisher Scientific). The relative change in mRNA expression was calculated using the 2^ΔΔCT^ method with GAPDH mRNA as an internal normalizer. All results are presented as the fold-increase in mRNA expression relative to LPS-treated cells.

### Isolation of detergent-insoluble plasma membrane fractions

BMDMs were transferred to SFM for 30 min and then treated with 10 nM EI-tPA, 10 nM α_2_M or vehicle for 1 h. Cells were washed three times with PBS, gently lifted from the plate, and then incubated with 2 mM EZ-Link Sulfo-NHS-LC-Biotin reagent (Thermo Fisher Scientific) for 30 min at 4 °C. This plasma membrane-impermeable reagent labels only the ectodomains of plasma membrane proteins. To quench biotinylation reactions, cells were extensively washed with 20 mM sodium phosphate, 150 mM NaCl, 100 mM glycine, pH 7.4. Cells were then extracted in 1% Triton X-100 containing protease inhibitors for 30 min at 4 °C. Extracts were centrifuged at 12,000× g for 20 min at 4 °C. Supernatants were collected and referred to as the Triton X-100-soluble fraction. The Triton X-100-insoluble pellets were re-extracted in RIPA buffer containing protease inhibitors for 30 min at 4 °C, then centrifuged at 12,000× g for 20 min at 4 °C. Biotinylated cell surface proteins from both fractions were affinity precipitated with Pierce Streptavidin Magnetic Beads (Thermo Fisher Scientific). Precipitates were subjected to SDS-PAGE and immunoblot analysis to detect LRP1 β-chain (Abcam), LRP1 α-chain (Sigma-Aldrich), GluN1 (Cell Signaling Technology), and PrP^C^ (POM19, a monoclonal antibody which is previously described^76^). Uncropped blots are presented in Supplementary Figures online.

### Statistics

Statistical analysis was performed using GraphPad Prism 9.3 (GraphPad Software). All results are expressed as the mean ± SEM. When “n” values are reported, each replicate was performed using a different macrophage preparation. Data were analyzed by one-way ANOVA followed by post-hoc Tukey’s multiple comparison test. *P*-values of *P < 0.05, **P < 0.01, ***P < 0.001, ****P < 0.0001 were considered statistically significant.

## Supplementary Information

Supplementary Information.

## Data Availability

The full-length images of the original western blot membranes are available online in Supplemental Figures S1 to S7. All data that support the findings of this study are available from the corresponding author on reasonable request.
