# Supplementary material for: The LRP1/CD91 ligands, tissue-type plasminogen activator, α2-macroglobulin, and soluble cellular prion protein have distinct co-receptor requirements for activation of cell-signaling
Source: Sci Rep. 2022 Oct 20;12:17594. doi: 10.1038/s41598-022-22498-1 (PMC9585055; doi:10.1038/s41598-022-22498-1)

The LRP1/CD91 ligands, tissue-type plasminogen activator, α_2-macroglobulin, and soluble cellular prion protein have distinct co-receptor requirements for activation of cell-signaling

Elisabetta Mantuano,[1]* Pardis Azmoon,[1] Michael A. Banki,[1] Cory B. Gunner,[1] and Steven L. Gonias[1]

[1]Department of Pathology, University of California San Diego, La Jolla, CA, 92093

*correspondence to E. Mantuano: emantuano@health.ucsd.edu

Supplementary Information:

- Figures S1 to S21

Fig. S1. Uncropped images of the western blot shown in Fig. 1A. The areas of the membrane that were cropped and presented in Fig. 1A are highlighted by red rectangles.

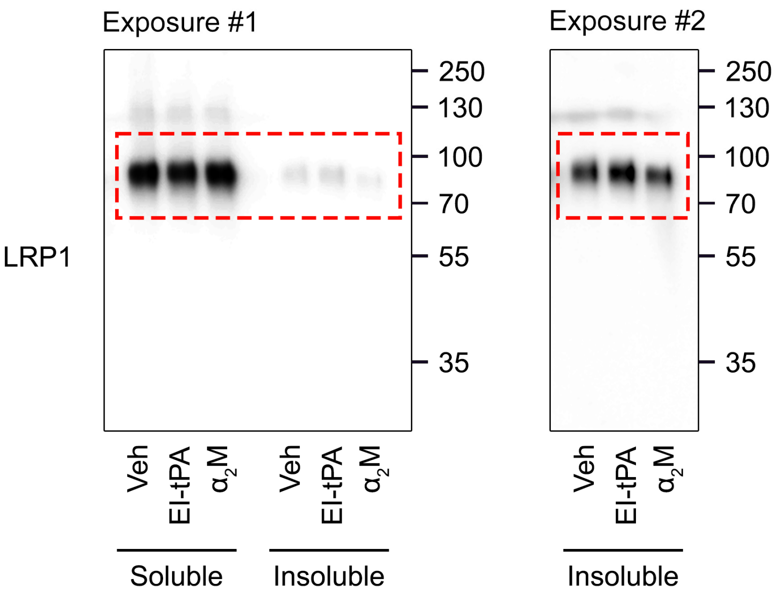

Fig. S2. Uncropped images of the western blot shown in Fig. 1B. The areas of the membrane that were cropped and presented in Fig. 1B are highlighted by red rectangles. Membranes were cut prior to hybridization with antibodies. Full-length blots showing the specificity of the single antibodies is shown in Fig. S20 for GluN1 and in Fig. S21 for PrP^{C}.

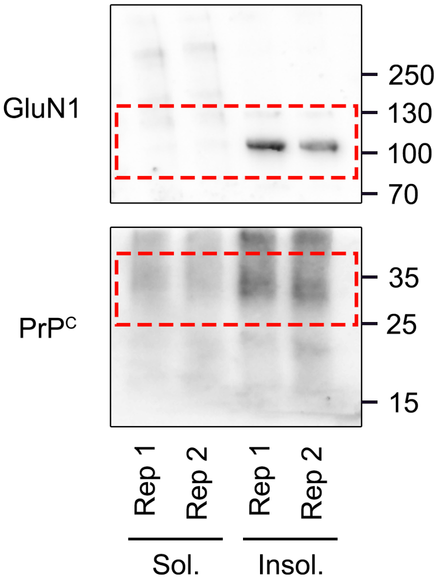

Fig. S3. Uncropped images of the western blot shown in Fig. 1D. The areas of the membrane that were cropped and presented in Fig. 1D are highlighted by red rectangles.

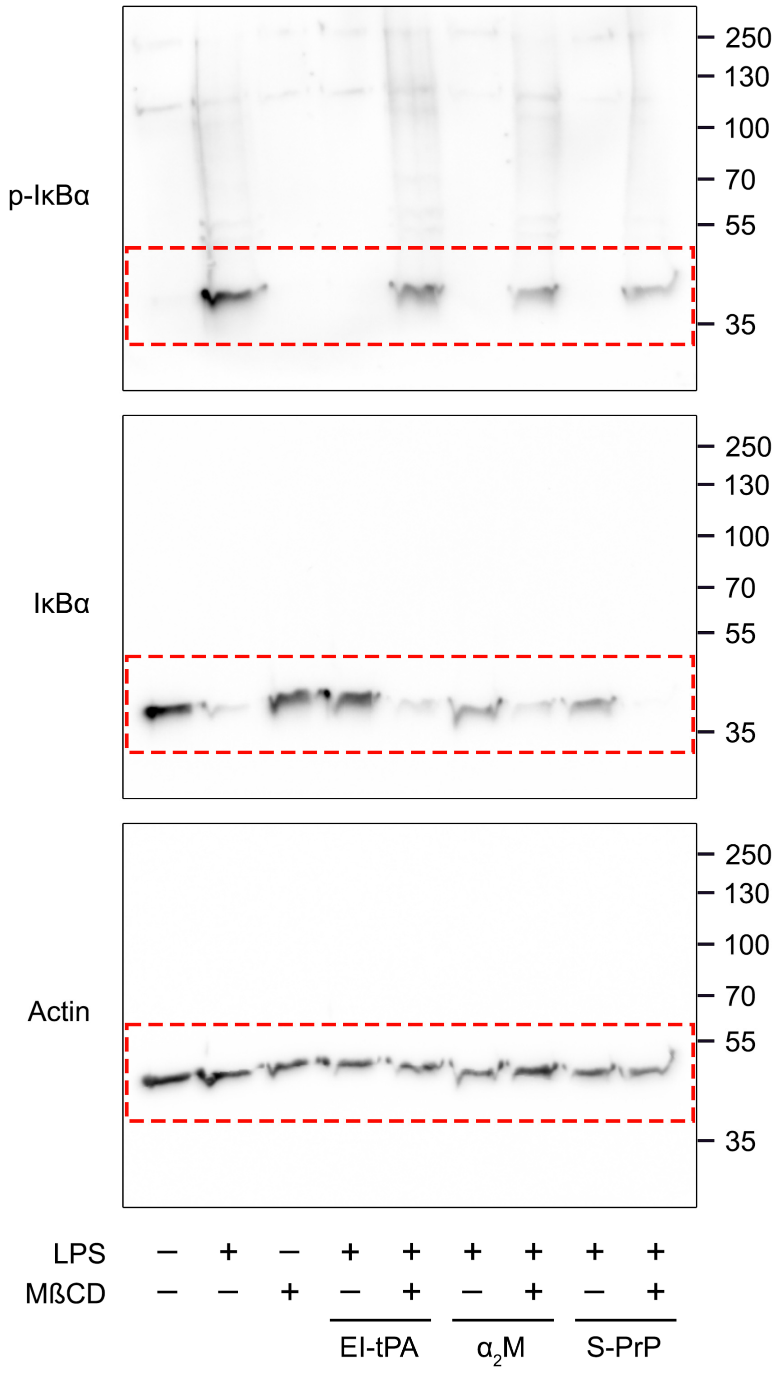

Fig. S4. Uncropped images of the western blot shown in Fig. 1E. The areas of the membrane that were cropped and presented in Fig. 1E are highlighted by red rectangles.

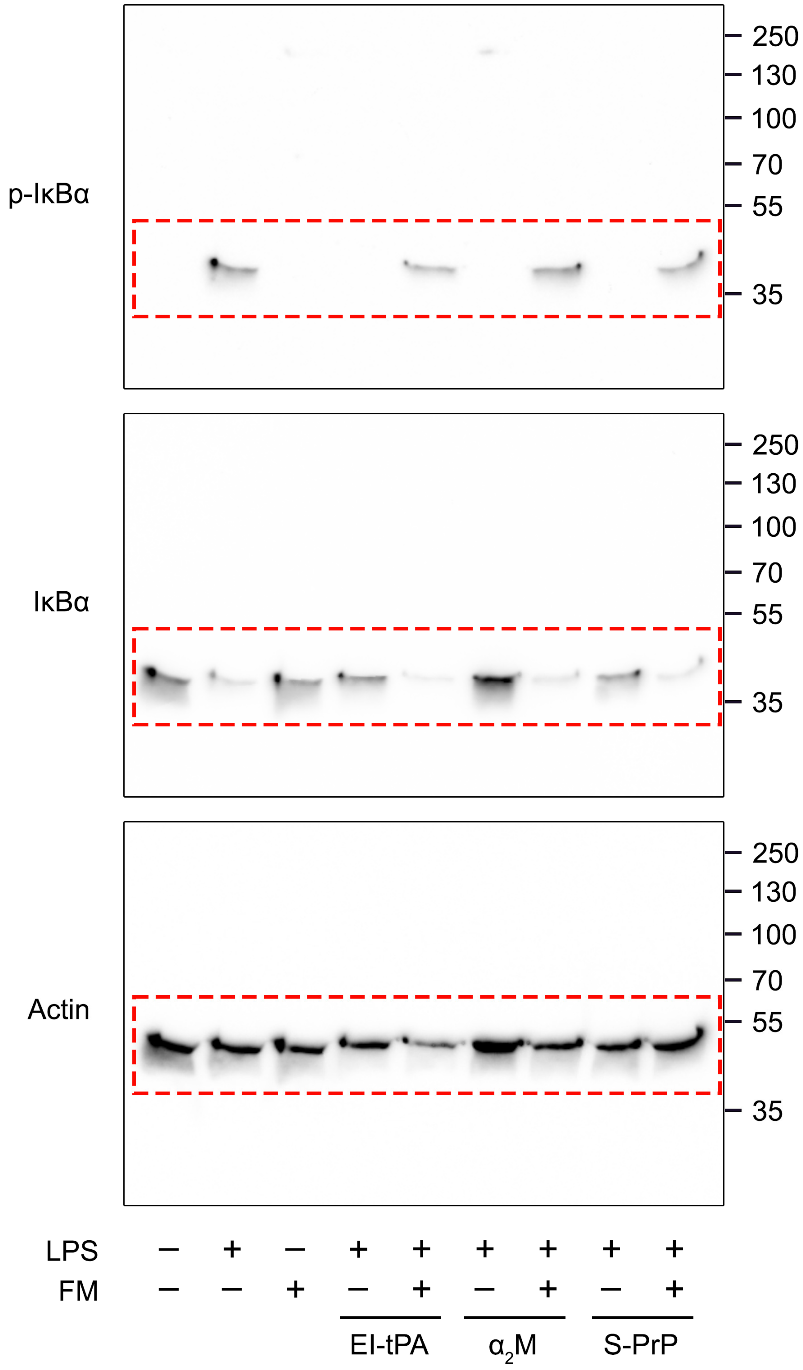

Fig. S5. Uncropped images of the western blot shown in Fig. 2A. The areas of the membrane that were cropped and presented in Fig. 2A are highlighted by red rectangles.

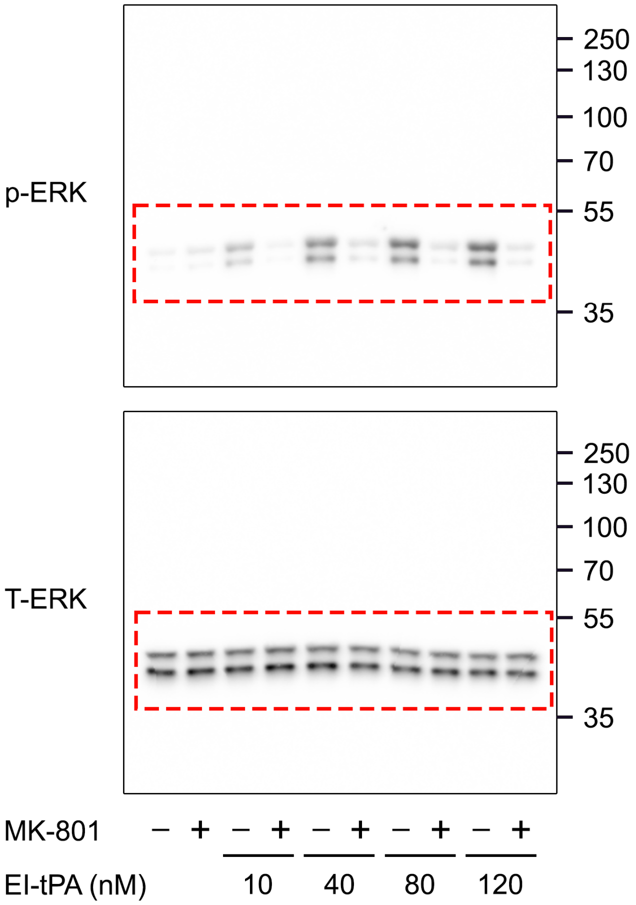

Fig. S6. Uncropped images of the western blot shown in Fig. 2C. The areas of the membrane that were cropped and presented in Fig. 2C are highlighted by red rectangles.

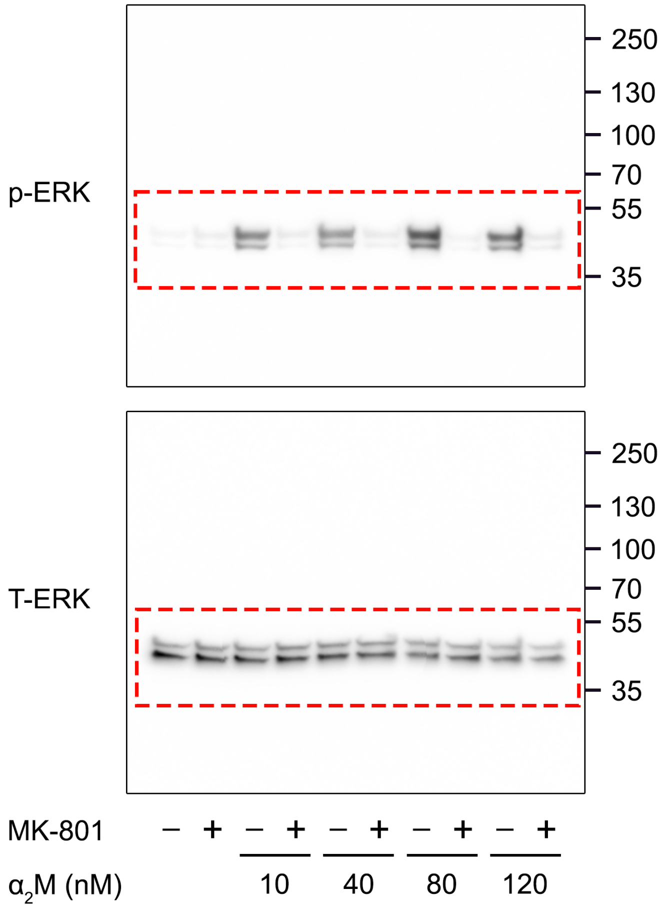

Fig. S7. Uncropped images of the western blot shown in Fig. 2E. The areas of the membrane that were cropped and presented in Fig. 2E are highlighted by red rectangles.

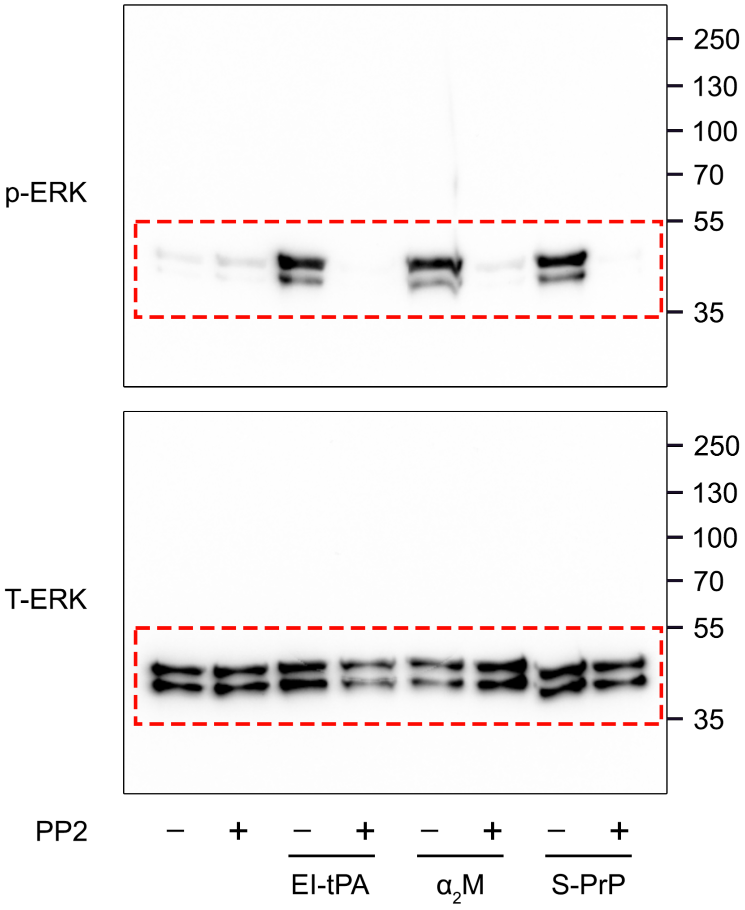

Fig. S8. Uncropped images of the western blot shown in Fig. 3A. The areas of the membrane that were cropped and presented in Fig. 3A are highlighted by red rectangles.

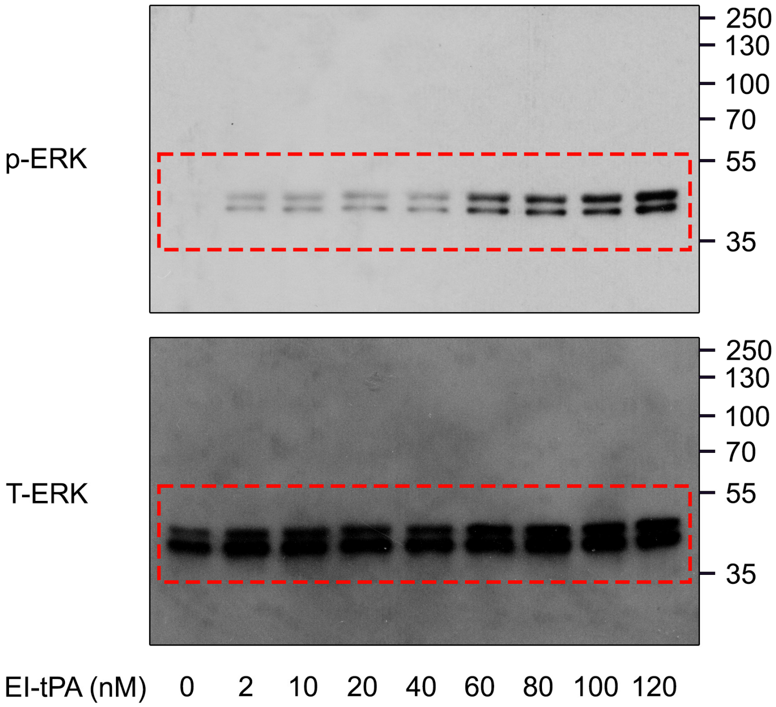

Fig. S9. Uncropped images of the western blot shown in Fig. 3B. The areas of the membrane that were cropped and presented in Fig. 3B are highlighted by red rectangles.

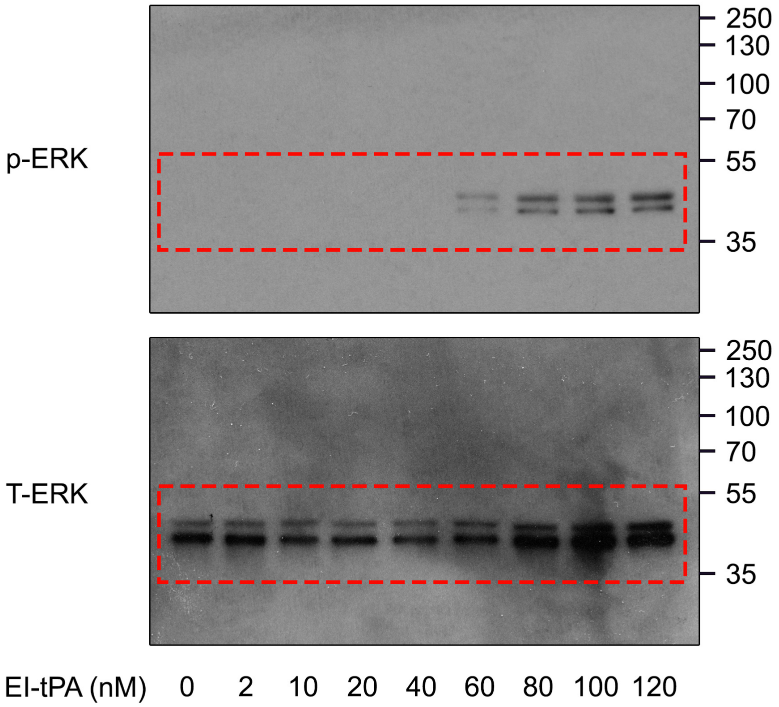

Fig. S10. Uncropped images of the western blot shown in Fig. 3C. The areas of the membrane that were cropped and presented in Fig. 3C are highlighted by red rectangles.

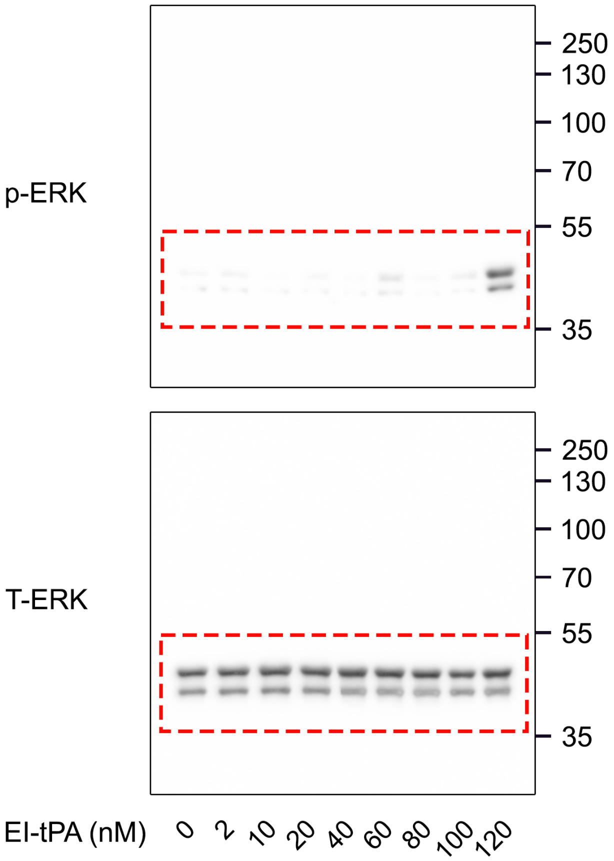

Fig. S11. Uncropped images of the western blot shown in Fig. 4A. The areas of the membrane that were cropped and presented in Fig. 4A are highlighted by red rectangles.

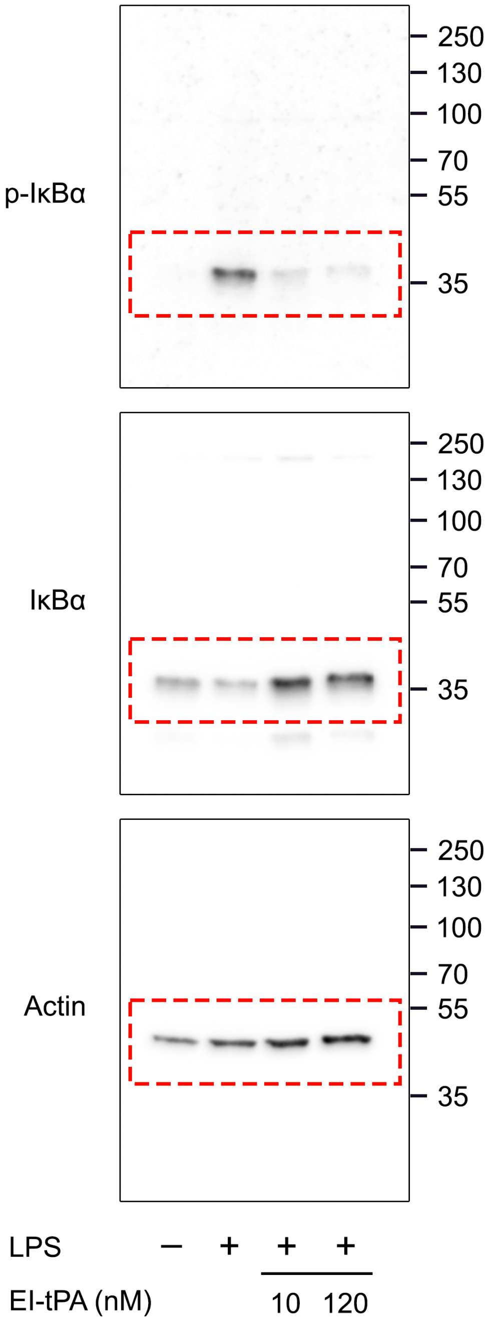

Fig. S12. Uncropped images of the western blot shown in Fig. 4C. The areas of the membrane that were cropped and presented in Fig. 4C are highlighted by red rectangles.

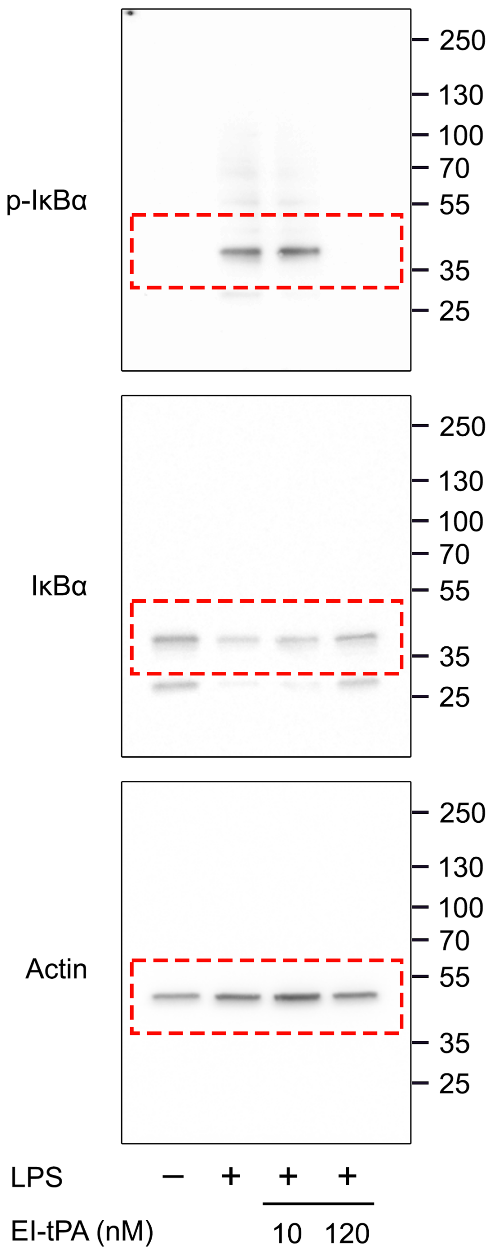

Fig. S13. Uncropped images of the western blot shown in Fig. 4E. The areas of the membrane that were cropped and presented in Fig. 4E are highlighted by red rectangles.

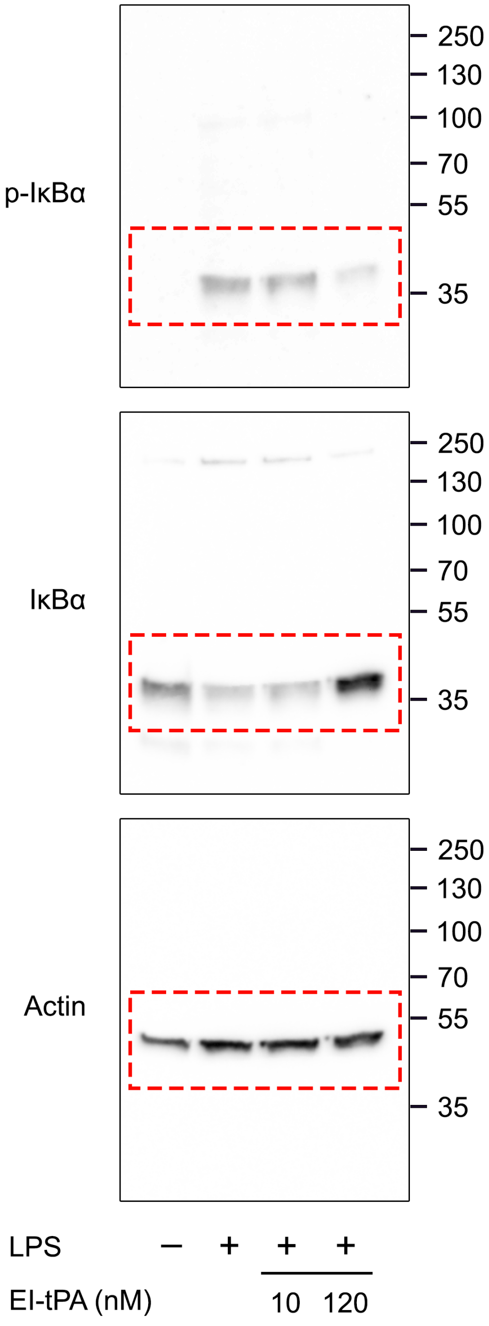

Fig. S14. Uncropped images of the western blot shown in Fig. 5A. The areas of the membrane that were cropped and presented in Fig. 5A are highlighted by red rectangles.

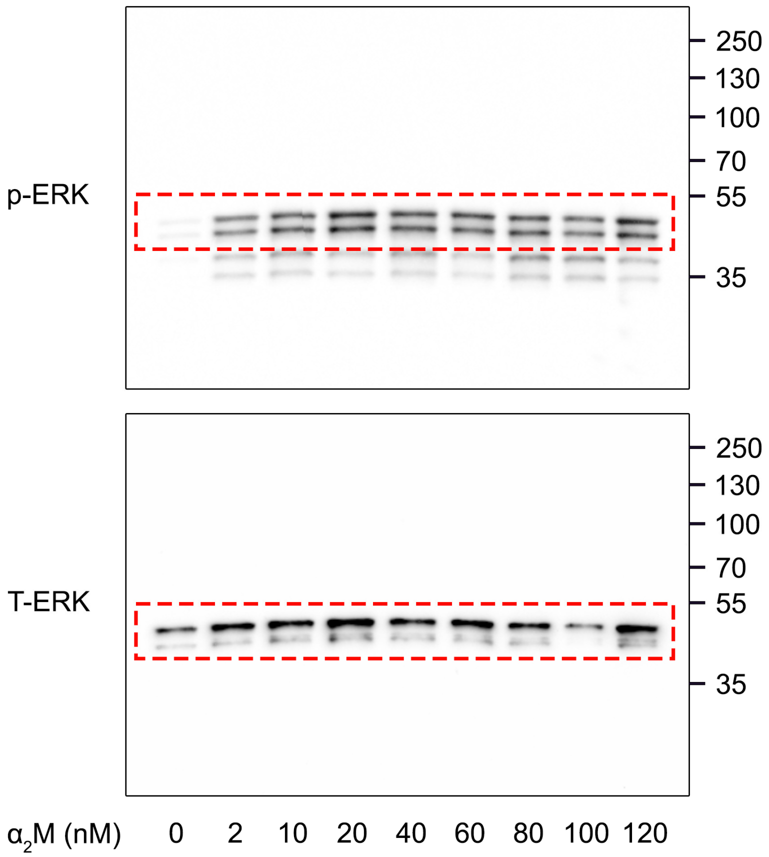

Fig. S15. Uncropped images of the western blot shown in Fig. 5B. The areas of the membrane that were cropped and presented in Fig. 5B are highlighted by red rectangles.

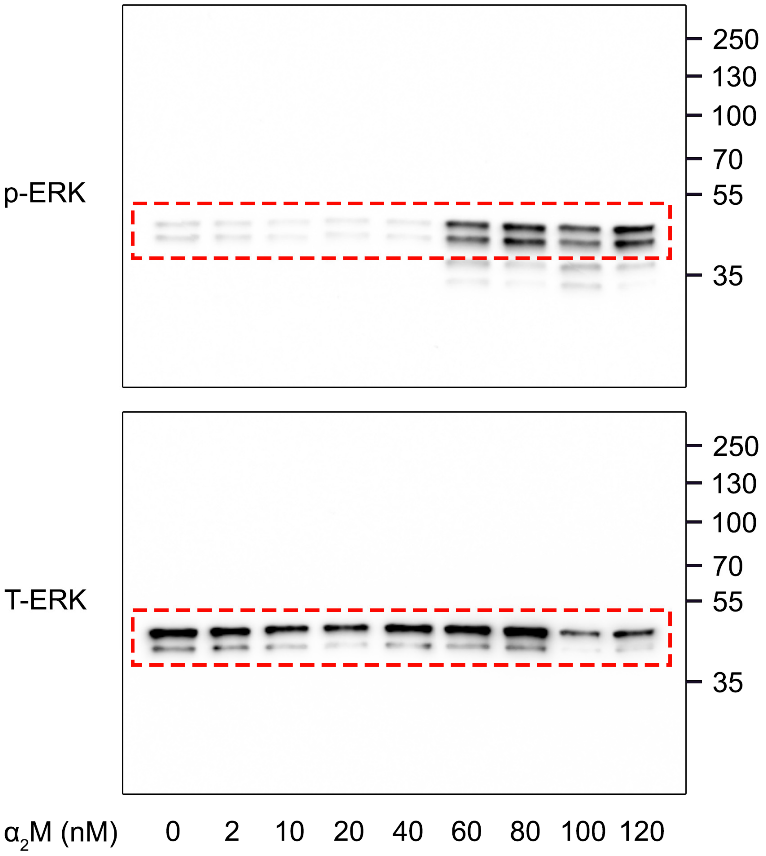

Fig. S16. Uncropped images of the western blot shown in Fig. 5C. The areas of the membrane that were cropped and presented in Fig. 5C are highlighted by red rectangles.

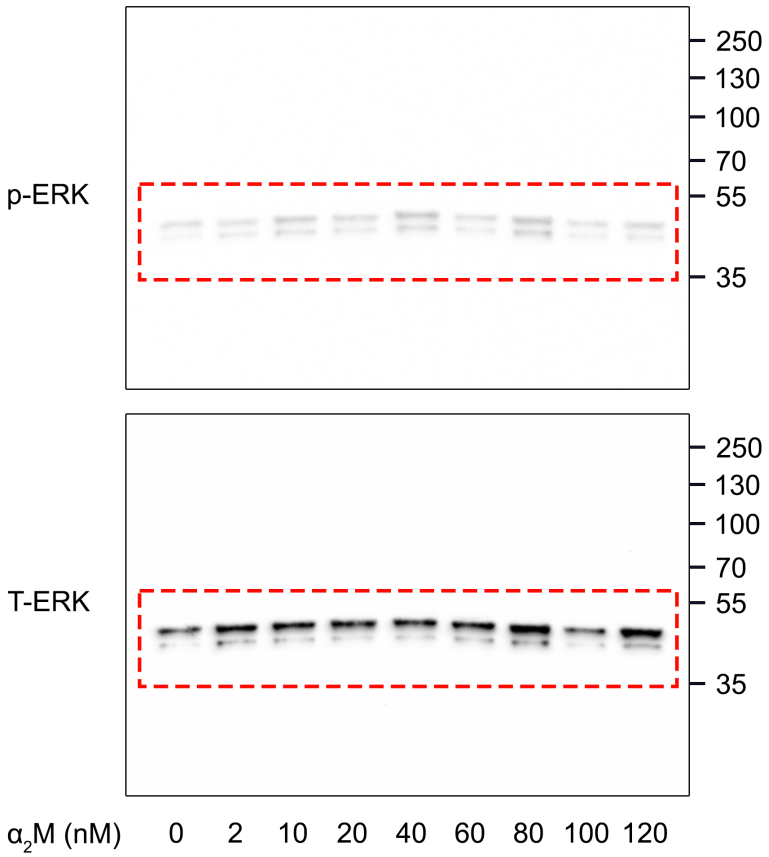

Fig. S17. Uncropped images of the western blot shown in Fig. 6A. The areas of the membrane that were cropped and presented in Fig. 6A are highlighted by red rectangles.

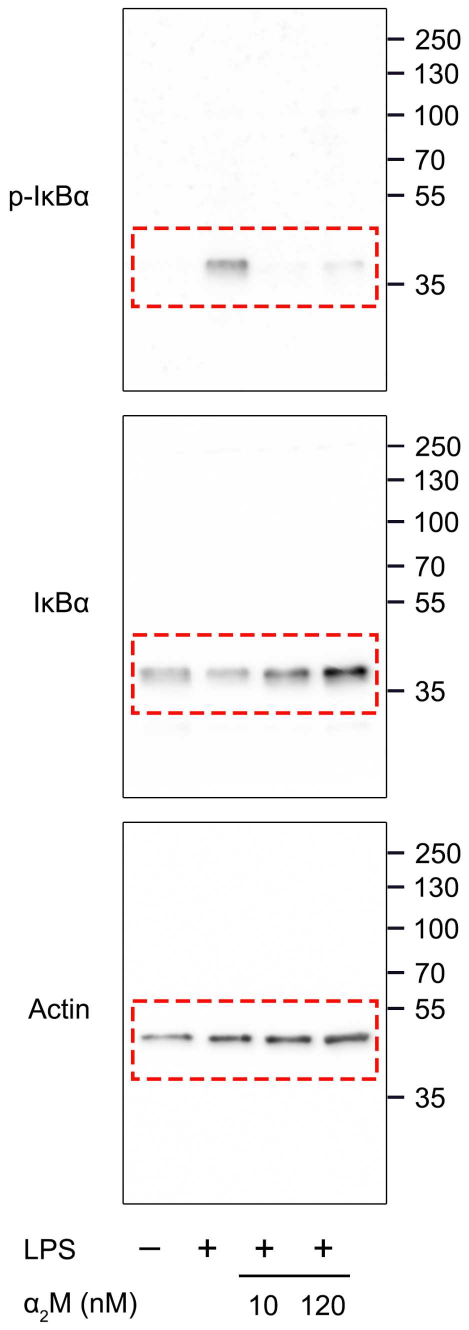

Fig. S18. Uncropped images of the western blot shown in Fig. 6C. The areas of the membrane that were cropped and presented in Fig. 6C are highlighted by red rectangles.

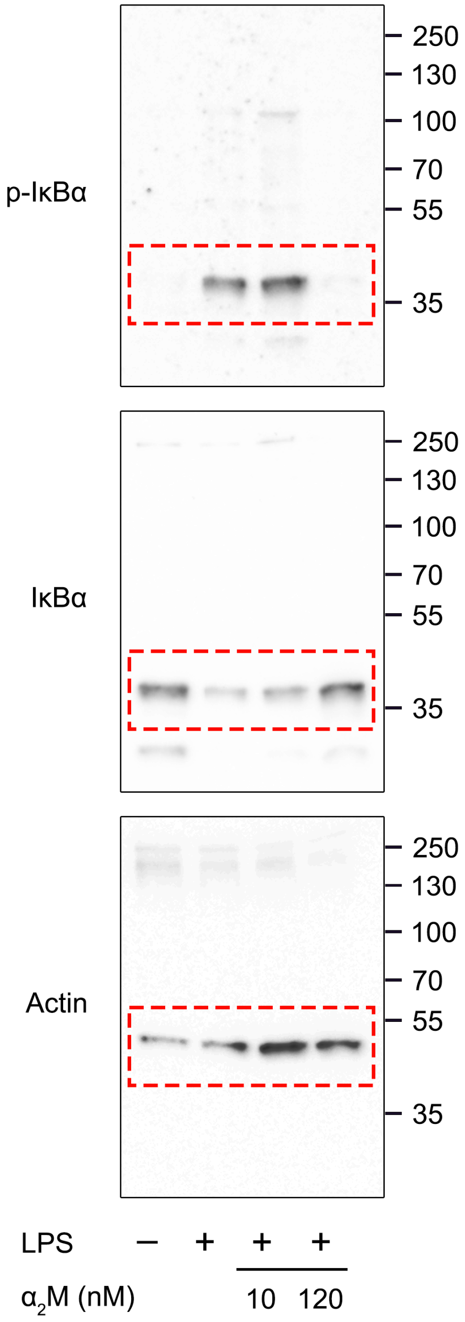

Fig. S19. Uncropped images of the western blot shown in Fig. 6E. The areas of the membrane that were cropped and presented in Fig. 6E are highlighted by red rectangles.

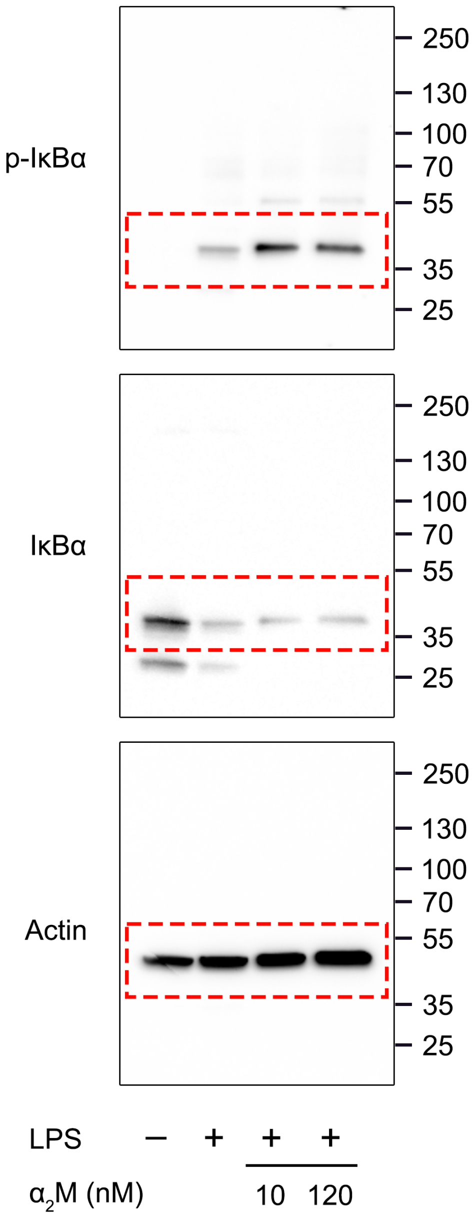

Fig. S20. Uncropped images of the western blot shown in Fig. 7. The areas of the membrane that were cropped and presented in Fig. 7 are highlighted by red rectangles.

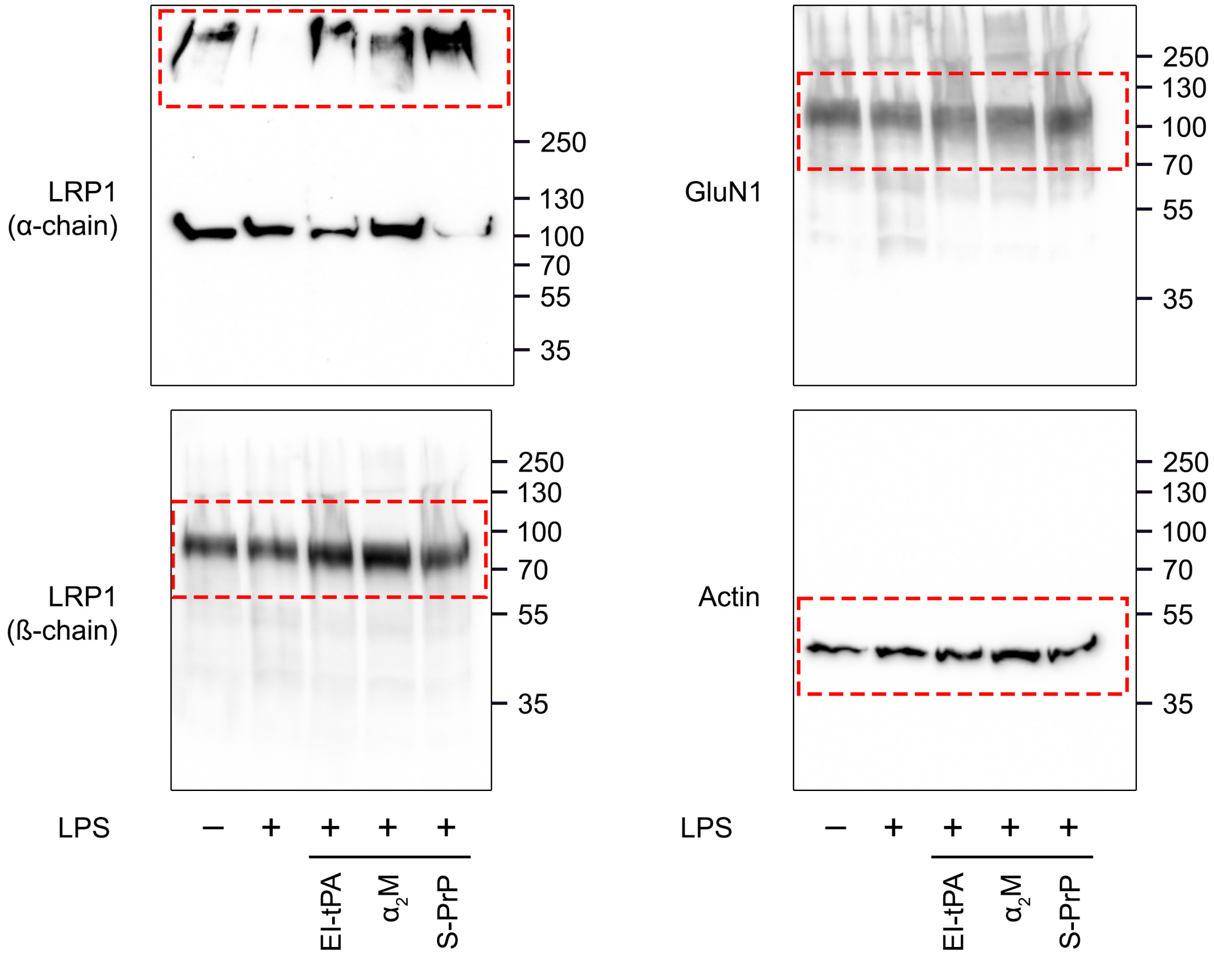

Fig. S21. Uncropped image of a western blot showing specificity for anti-PrP^C. BMDMs isolated from WT mice were extracted with RIPA buffer and subjected to immunoblot analysis to detect PrP^C.

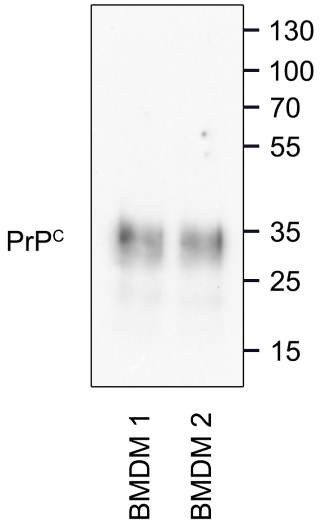

Supplement: Supplementary file 1 — Supplementary Information. [file 41598_2022_22498_MOESM1_ESM.pdf]
